# Heavy Metals Toxicity: Mechanism, Health Effects, and Therapeutic Interventions

**DOI:** 10.1002/mco2.70241

**Published:** 2025-08-19

**Authors:** Yu‐feng Cheng, Yu‐jia Zhao, Ce Chen, Feng Zhang

**Affiliations:** ^1^ Key Laboratory of Basic Pharmacology of Ministry of Education and Joint International Research Laboratory of Ethnomedicine of Ministry of Education and Key Laboratory of Basic Pharmacology of Guizhou Province and Laboratory Animal Centre Zunyi Medical University Zunyi Guizhou China; ^2^ Sino‐Jan Joint Lab of Natural Health Products Research School of Traditional Chinese Pharmacy China Pharmaceutical University Nanjing China

**Keywords:** disease, heavy metal toxicity, mechanism, therapeutic intervention

## Abstract

Heavy metals (HMs), such as chromium, arsenic, cadmium, mercury, and lead, constitute a class of environmental pollutants with significant toxicity that pose a serious threat to human health. This review provides a comprehensive overview of the biochemical properties of HMs, and their effects at the cellular, molecular, and genetic levels. HMs exert their toxic effects by interfering with various intracellular biochemical processes, including enzyme activity, protein synthesis, and energy metabolism. Furthermore, they can disrupt the integrity of cell membranes and affect cellular signaling, leading to cellular dysfunction and death. At the molecular and genetic levels, HMs can cause DNA damage and induce gene mutations, thereby affecting genetic transmission and expression. Then, the effects of HMs on the nervous system, kidneys, cardiovascular system, reproduction, and cancer risk are discussed. Therapeutic strategies, such as chelation therapy, antioxidants and free radical scavengers, supportive therapy, and prevention and reduction of exposure, have been shown to mitigate the toxic effects of HMs. Last, based on the current findings on the mechanisms of HMs, future research directions are prospected. Through multidisciplinary cooperation and integrated interventions, it is expected that the health risks posed by HMs can be alleviated. Future research needs to further elucidate the mechanisms of HMs toxicity, develop more effective treatments, and strengthen preventive and control measures.

## Graphical abstract

Heavy metals exert a wide range of effects at the cellular, molecular, and genetic levels, leading to neurological damage, renal dysfunction, cardiovascular disease, reproductive developmental abnormality, immune system abnormality, and increased risk of cancer by interfering with biomolecule function, signaling pathways, inducing oxidative stress, and inhibiting enzyme activity, among other mechanisms. Therapeutic interventions for heavy metals include chelation therapy, antioxidant application, supportive therapy, and exposure reduction strategies aimed at mitigating the toxic effects of heavy metals. Future research is needed to further elucidate the mechanisms of toxicity, develop more effective therapeutic approaches, and strengthen environmental management and public health education in order to minimize the risks of heavy metals to human health.

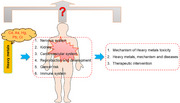

## 1 Introduction

Heavy metals (HMs) have long been regarded as important environmental pollutants due to their toxicity and persistence in ecosystems. Historically, the adverse effects of HMs on human health and the environment have been documented for centuries. For example, the dangers of lead (Pb) poisoning were known in ancient Rome, and mercury (Hg) toxicity had been observed in populations consuming contaminated fish [1, 2]. In Japan, Hg poisoning has also sparked widespread global concern about the toxicity of HMs [3]. The use of traditional medicine with certain HMs‐based drugs, such as cinnabar and realgar, has also triggered the investigation of HMs toxicity. Cinnabar, the main component is HgS, is used in traditional medicine for its purported calming effects on the nervous system and other therapeutic purposes [4, 5, 6, 7].

With the rapid development of modern industrialization and urbanization, the problem of HMs pollution is becoming serious. Various factors, including industrial emissions, agricultural activities, mining extraction, and atmospheric deposition, have led to widespread contamination of soil, water, and air with HMs, such as chromium (Cr), arsenic (As), cadmium (Cd), Hg, and Pb [8]. These metals can accumulate through the food chain and pose a serious threat to human health through a variety of pathways, including intake, inhalation, and dermal contact [9]. Current research focuses on understanding the mechanisms of HMs toxicity, assessing their health effects, and developing effective alleviation and prevention strategies.

The serious problem of HMs pollution and its widespread impact on human health highlights the necessity of a comprehensive analysis of the biochemical properties and toxicity mechanisms of HMs. This process not only fills gaps in our existing knowledge, but also promotes the development of therapeutic and preventive strategies. Additionally, it provides an important basis for the formulation of public health policies and the guidance for future research directions. Given the complexity and multidisciplinary nature of the HMs pollution problem, this review was conceived with the objective of integrating and organizing the latest research findings on the mechanisms of HMs toxicity, health effects, and intervention strategies. By systematically summarizing the sources, pollution pathways, toxicity mechanisms, and effects of HMs on different organ systems, this review not only enhances the understanding of HMs pollution problems, but also promotes interdisciplinary cooperation and the formulation of more effective pollution prevention strategies and health protection measures. Furthermore, this review aims to reveal the shortcomings in existing research and propose potential avenues for future investigation, with the aim of offering inspiration and guidance for subsequent scientific studies, so as to better address the global challenges posed by HMs pollution.

Here, this review introduces HMs definition, classification, sources, and their pollution pathways. Next, the mechanisms underlying HMs toxicity are explored in detail, including their interactions with biomolecules, induction of oxidative stress (OS), inhibition of enzyme activities, and interference with metabolic pathways. Subsequently, the effects of HMs on different organ systems and their roles in the development of diseases are analyzed. Then, current treatment strategies and preventive measures are discussed. Finally, the key findings of the review are summarized, and future research directions are prospected.

## 2 Heavy Metals

### 2.1 Definition and Classification of HMs

HMs can be defined as metals with atomic number over 20 and a density exceeding 5 g/cm^3^. These metals can cause chronic toxicity when they accumulate to a certain extent in the human body [10, 11]. Moreover, HMs can be classified into four categories based on their environmental and biological impacts. Category (I): highly toxic HMs, including Pb, Hg, and Cd, pose significant threats to human health and the ecological environment, and their use and emissions should be strictly controlled. Category (II): moderately toxic HMs, including Cr, cobalt, and manganese, which are harmful to human health and the ecological environment, necessitating monitoring. Category (III): low‐toxicity HMs, including copper (Cu), zinc (Zn) and iron, pose relatively less harmful to human health and the ecosystem, but can also have an impact on the environment if accumulated excessively. Category (IV): trace HMs, including molybdenum (Mo), vanadium (V), antimony (Sb), and so on, although minimal risk to human health and the ecosystem environment, also require monitoring and controlling.

### 2.2 Sources and Ways of HMs Pollution

The sources and ways of HMs pollution are crucial topics in environmental science, with four main categories identified. Category (I): Industrial emissions are the main sources of HMs pollution. With the rapid development of industrialization and urbanization, the potential impact of HMs pollution on the environment and human health is increasingly a matter of public concern [12]. Category (II): Agricultural practices where pesticides and fertilizers use contribute to HMs contamination of soils, affecting their distribution in the soil [13]. Category (III): Mining activities,  mining and smelting activities release large quantities of toxic and harmful HMs into the soil, causing serious soil HMs pollution [14]. Category (IV): Atmospheric deposition, where HMs in the atmosphere can enter soil and water through aerosol deposition [15].

Correspondingly, there are three main ways in which HMs affect human health. Category (I): *Soil pollution*: soil is the main conduit for HMs pollution. Once HMs infiltrate the soil via different routes, they will affect the growth of microorganisms and plants and enter the human body through the food chain, and after accumulating to a certain extent, they will damage multiple organs and systems, and threaten human health. Category (II): *Water pollution*: when HMs enter water environments through industrial wastewater and agricultural drainage, affecting water quality and human safety. Category (III): *Atmospheric pollution*: HMs enter the atmosphere mainly through industrial emissions and traffic exhaust and then enter the human body through the respiratory system or affect soil and water through deposition.

In summary, HMs are derived from diverse sources, including industrial emissions, agricultural activities, and natural geological processes, and these pollutants ultimately enter the human body through the food chain, drinking water, and direct exposure, causing multisystemic harm health effects.

## 3 Mechanism of HMs Toxicity

HMs exert toxic effects through multiple mechanisms, mainly including interactions with biomolecules, induction of OS and reactive oxygen species (ROS) production, inhibition of enzymatic activity, and interference with metabolic pathways. These mechanisms are correlated and lead to cellular dysfunction and damage. Binding of HMs to proteins and nucleic acids directly impaired their function, while ROS production further exacerbated cellular damage. In addition, HMs inhibited the activity of key enzymes, which could lead to metabolic and intracellular homeostatic disorders. In‐depth understanding of these mechanisms and their interrelationships is essential for elucidating the toxicological properties of HMs.

### 3.1 Biochemistry of HMs

#### 3.1.1 Interaction with Biomacromolecule

Biomacromolecules, including proteins, nucleic acids, and polysaccharides, are the basic substances of living organisms and perform important functions in the body [16, 17]. Hexavalent Cr (VI) had the potential to disrupt chromatin structure through direct oxidation and the formation of Cr–DNA complexes, a process that triggered the DNA damage response and facilitated the chromatin structural adjustments necessary for DNA repair mechanisms [18]. As interferes with protein function by binding to cysteine residues in proteins and forming complexes with thiols that produce toxic effects [19, 20, 21]. Interestingly, one mechanism by which HMs ions interfered with protein function is through ionic mimicry, wherein they could minic other ions, which is known as. For example, Cd could mimic native metal ions, such as calcium and Zn, by competing with protein binding sites, potentially altering protein structure and function [22]. Similarly, Hg could interact with sulfur groups in proteins, resulting in protein structural changes and function loss, which could lead to OS [23]. Additionally, Pb (II) could bind to sulfhydryl groups in proteins, leading to alterations in protein structure and function. Specially, the enzyme δ‐aminolevulinic acid dehydratase (ALAD) contained a Zn(II)–Cys3 site that could be replaced by Pb [24]. These mechanisms reveal the potential hazards posed by HMs to organisms and highlight their disruptive effects on the normal functions of biomacromolecules.

#### 3.1.2 OS and ROS

Humans are primarily exposed to two oxidation states of Cr: trivalent Cr (Cr (III)) and Cr (VI). Notably, ROS were generated during the reduction of Cr (VI) to Cr (III), leading to OS and DNA damage. Furthermore, Cr (VI) was often present in the environment as the chromate anion. Due to its structural similarity to sulfate, it could enter the cells via the cellular sulfate transporter protein. Once inside the cells, Cr (VI) reacted with ascorbic acid and thiols, such as glutathione (GSH), producing hydrogen peroxide (H_2_O_2_) and free radicals, triggering OS, and causing damage to lipids, proteins, and DNA [25]. Likewise, under physiological conditions, As predominantly exists in the As (III) and pentavalent As (V), with As (III) exhibiting higher toxicity. As (III) could be oxidized to As (V) by redox reactions, and the methylation process was a crucial step in As metabolism, generating intermediates and potentially ROS. In addition, As (V) could be reduced back to As (III), leading to ROS generation and OS [26]. Cd‐induced OS was associated with its activation of nicotinamide adenine dinucleotide phosphate oxidase (NADPH) oxidase (NOX), an enzyme complex found in a wide range of cell types that primarily facilitated the transfer of electrons from reduced NADPH to oxygen molecules, resulting in the production of ROS [27]. Chronic exposure to Hg increased extracellular glutamate levels, potentially due to enhanced glutamate release and/or decreased uptake, culminating in the overproduction of free radicals, such as nitric oxide (NO) [28]. Furthermore, Pb could directly stimulate the production of ROS, including mono‐linear oxygen (^1^O_2_), H_2_O_2_, and hydroxyl radical (OH^−^) [29]. These observations indicated that HMs, such as Cr, As, Cd, Hg, and Pb, share the common property of generating ROS and triggering OS through distinct mechanisms, which might ultimately lead to cellular damage and a variety of health issues.

#### 3.1.3 Inhibition of Enzyme Activity and Interference of Metabolic Pathways

**FIGURE 1:**
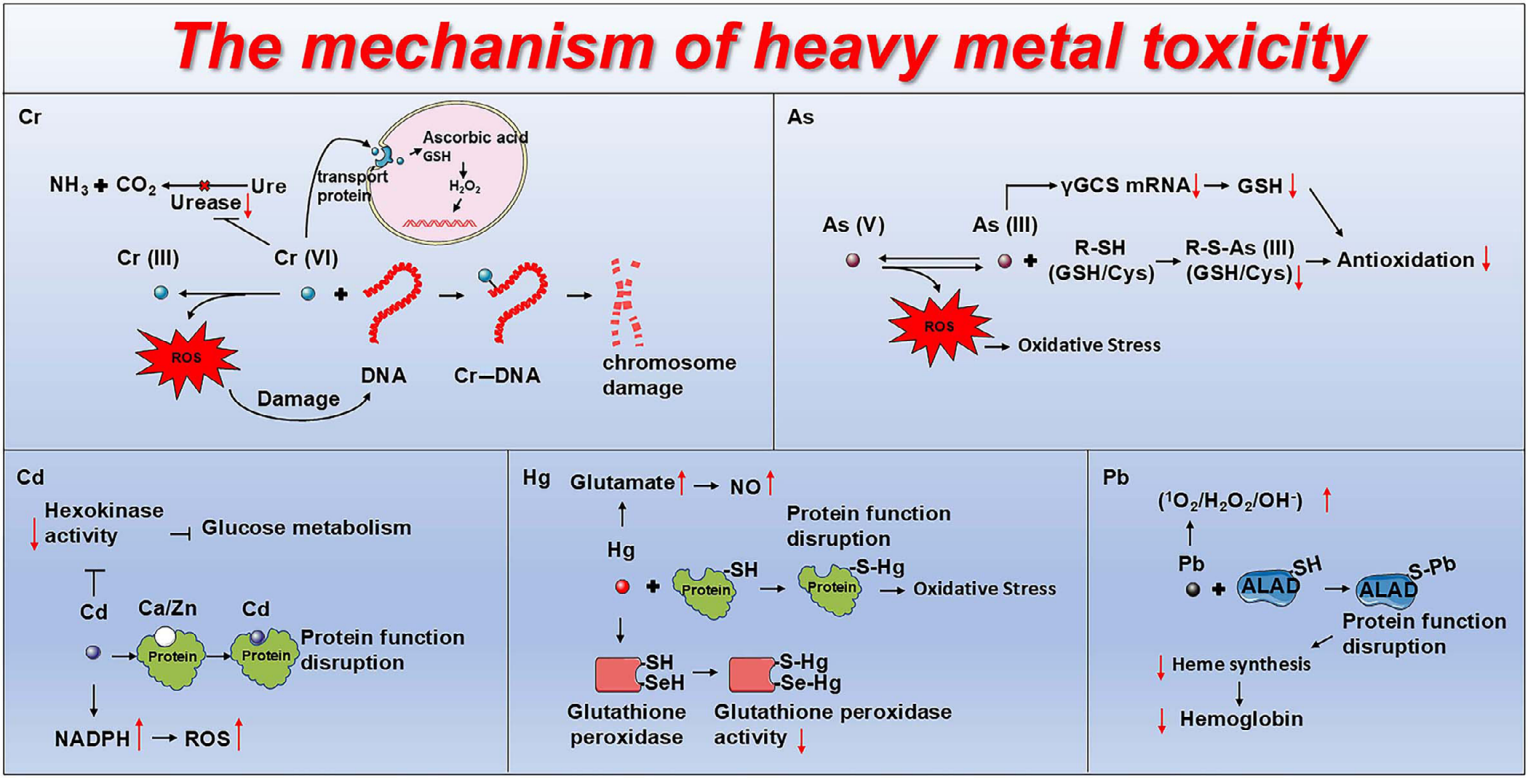
The mechanisms of HMs tocicity. Cr: Cr (III) can be oxidized to Cr (VI), which forms Cr–DNA complexes, resulting in DNA and chromosomal damage. Cr (VI) enters cells via sulfate transporters and reacts with ascorbic acid and GSH to produce ROS, causing OS‐induced DNA damage. Furthermore, Cr (VI) inhibits urease activity in nitrogen metabolism, affecting urea hydrolysis. As: Reduction of As(V) to As(III) generates ROS, leading to OS. As (III) binds to the thiol groups in GSH and cysteine, reducing the cellular antioxidant capacity. Additionally, As (III) decreases the mRNA levels of γGCS, which further reduces GSH levels and diminishes the cellular antioxidant capacity. Cd: Cd competes with calcium and zinc binding sites on proteins, thereby disrupting protein function. Cd inhibits hexokinase activity, affecting glucose metabolism, and generates ROS by activating NADPH oxidase activity. Hg: Hg binds to thiol groups in proteins, leading to protein dysfunction and OS. Hg increases glutamate levels, causing excessive production of NO, which further induces OS. Additionally, Hg binds to thiol and selenol groups in glutathione peroxidase, leading to decreased enzymatic activity. Pb: Pb induces ROS production, including ^1^O_2_, H_2_O_2_, and OH^−^, leading to OS. Pb binds to the thiol groups in ALAD, inhibiting its function, which further suppresses heme synthesis and reduces hemoglobin levels. γGCS, γ‐glutamylcysteine synthetase; ALAD, δ‐aminolevulinic acid dehydratase; As, arsenic; Cd, cadmium; Cr, chromium; Cys, cysteine; GSH, glutathione; Hb, hemoglobin; Hg, mercury; NADPH, nicotinamide adenine dinucleotide phosphate; NO, nitric oxide; Pb, lead; ROS, reactive oxygen species.

Urease is a pivotal enzyme involved in nitrogen metabolism, which plays a crucial role in the urea cycle by catalyzing the hydrolysis of urea into ammonia and carbon dioxide. Notably, elevated concentrations of Cr inhibited the activity of urease, and interfered with the normal process of urea decomposition [30]. Exposure to AS (III) also reduced the mRNA and protein expression levels of γ‐glutamylcysteine synthetase (γGCS), leading to reduced synthesis of GSH and impaired cellular antioxidant capacity [31]. Cd exposure significantly diminished the activity of hexokinase of the glycolytic pathway in the cardiac tissue, thereby affecting glucose metabolism and lipid accumulation [32]. Hg interacted with the thiol and selenol groups of the GSH peroxidase that resulted in reduced enzymatic activity [33]. Pb (II) inhibited the activity of ALAD, thereby obstructing heme synthesis and causing hemoglobin deficiency [24]. HMs interfered with the activity of key enzymes across critical metabolic pathways, including nitrogen metabolism, glucose metabolism, antioxidant systems, and heme synthesis, thereby negatively affecting cellular and organismal functions (Figure 1).

### 3.2 Effects at the Cellular Level

#### 3.2.1 Cell Membrane Damage

Cr (VI) could alter cell membrane permeability, impair membrane integrity, and decrease the expression of the tight junction proteins, including zonula occludens 1 and occludin, leading to membrane dysfunction [34]. When bound to sphingomyelin (SM), a crucial component of cell membranes, As (V) triggered SM polymerization, and increased membrane permeability [35]. Cd was recognized as a membrane toxicant that was capable of interfering with ion channels and transporter proteins in cell membranes, causing alterations to membrane structure and function [36]. Cell membranes were primary targets of Hg‐induced damage, and Hg could disrupt the physiological phospholipid asymmetry in erythrocytes, and affect ankyrin and flotillin‐2 protein expression, leading to diminished membrane stability and increased fragmentation [37].

#### 3.2.2 Mitochondrial Dysfunction

Exposure to Cr (VI) significantly increased the production of ROS and decreased the mitochondrial membrane potential (MMP), potentially causing abnormal mitochondrial structural changes, such as swelling and vacuoles formation [38]. Chronic As exposure has been shown to significantly decrease the mRNA and protein expression of the mitochondrial transcriptional coactivator peroxisome proliferator‐activated receptor gamma, coactivator 1α and its downstream target nuclear respiratory factors 1 (NRF1), NRF2, and transcription factor A mitochondrial, suggesting mitochondria damage [39]. Cd toxicity primarily raised from its inhibition of the mitochondrial electron transport chain (ETC), with a pronounced sensitivity to complex III, leading to its inhibition by approximately 77% [40]. Methylmercury (MeHg) might increase the release of calcium ions from the mitochondria into the cytoplasm by increasing mitochondrial membrane permeability, which could facilitate pyruvate entry into the mitochondria, thereby exacerbating the toxic effects of MeHg [41]. Pb inhibited the mitochondrial ETC, particularly at complex III, hindering electron flow and consequently diminishing the ability to synthesize adenosine triphosphate (ATP) [42].

#### 3.2.3 Interference with Cellular Signal Transduction

Cr (VI)‐induced OS, lipid accumulation, and glycemic abnormalities were associated with dysregulation of the ROS/Nrf2/heme oxygenase‐1 (HO‐1) signaling pathway [43]. As activated the NF‐κB signaling pathway and enhanced the expression of vascular cell adhesion molecule‐1 triggered by tumor necrosis factor‐α (TNF‐α), subsequently activating proinflammatory cytokines and eliciting cellular inflammatory responses [44]. Cd might induce cellular pyroptosis by provoking mitochondrial OS and activating the cGAS–STING signaling pathway [45]. Hg‐induced OS initiated apoptosis and necrosis by activating the p38 mitogen‐activated protein kinase (MAPK) signaling pathway [46]. Pb exposure activated death receptor pathways, notably the Fas/FasL system, which subsequently triggered the caspase cascade, leading to apoptosis [47].

### 3.3 Impacts at the Molecular and Genetic Levels

#### 3.3.1 DNA Damage and Genetic Mutations

Both Cr (III) and Cr (VI) significantly induced gene mutations and DNA damage, albeit through distinct mechanism. Cr(III) caused damage by disrupting DNA base stacking patterns and inducing DNA breaks, whereas Cr(VI) altered DNA structural changes by intercalating within the DNA plane [48]. As exposure induces OS, resulting in DNA damage and the inhibition of sensitive Zn finger DNA repair proteins. Although As itself was not a potent mutagen, it could significantly enhance the mutagenic effects of other mutagens, such as ultraviolet radiation and chemicals, a phenomenon known as “synergistic carcinogenesis” [49]. Similarly, low concentrations of Cd significantly increased the risk of genetic mutations and in turn promoted carcinogenesis by inducing OS and inhibiting DNA repair system [50]. MeHg and mercuric chloride (HgCl_2_) significantly increased DNA damage, especially affecting mitochondrial DNA and nuclear DNA. Hg was also capable of inhibiting the activity of DNA repair systems, particularly the base excision repair (BER) and nucleotide excision repair (NER) pathways [51]. Likewise, Pb exposure resulted in the reduced expression of DNA repair genes in the BER, NER, and double‐strand break repair pathways [52].

#### 3.3.2 Epigenetic Modifications

Cr (VI) exposure repressed the expression of genes such as O 6‐methylguanine–DNA methyltransferase (DNMT), mutl homolog 1, and semaphorin 4B by inducing hypermethylation of their promoter regions, and these gene downregulation was closely associated with cellular carcinogenesis, impaired DNA repair and metabolic disorders [53, 54, 55]. As (V) and As(III) were important environmental carcinogens that influenced the methylation status of cellular DNA [56]. The metabolism of As in organisms required substantial amounts of S‐adenosylmethionine (SAM), a crucial intracellular methyl donor. Depletion of SAM reduced the availability of methyl groups necessary for DNA methylation, which might lead to gene hypomethylations. This hypomethylation adversely affected gene expression, which in turn increased the risk of disease development [57, 58]. Additionally, As exposure had been shown to cause significant alterations in histone modifications, particularly histone methylation. Specifically, As exposure caused changes in histone H3 lysine 4 trimethylation (H3K4me3) and H3 lysine 27 trimethylation (H3K27me3) levels, that are crucial in the regulating gene expression [59]. Notably, H3K4me3 was typically associated with gene activation, whereas H3K27me3 was linked to gene repression [60, 61]. In mammalian kidneys, Cd exposure decreased DNA methylation levels due to the inhibition of DNMT activity, thereby affecting gene expression [62]. Furthermore, Cd exposure inhibited histone deacetylase (HDAC) activity, leading to increased histone acetylation and altered chromatin structure, which subsequently influenced the gene transcriptional activity [63]. Hg exposure also affected epigenetic modifications and altered gene expression patterns, thereby regulating neurological development [64]. Mechanisms underlying Hg‐induced brain tumors included hypomethylation of specific genes and hypermethylation of CpG sites, which might lead to dysregulated gene expression and tumor development [65]. Chronic Pb exposure resulted in significantly elevated histone acetylation levels in hippocampal tissues, potentially linked to the enzymatic activity of histone acetyltransferase p300. Notably, the transcriptional level of p300 was significantly elevated following high‐dose Pb exposure [66].

#### 3.3.3 Regulation of the Cell Cycle and Apoptosis

Cr (VI)‐induced cell cycle arrest was primarily mediated through a p53‐dependent pathway, wherein p53 protein was activated in response to stressors, such as DNA damage, which in turn regulated the expression of cell cycle‐inhibitory genes (e.g., p21 and p27). Concurrently, Cr(VI)‐induced apoptosis predominantly occurred via the mitochondrial pathway, characterized by the downregulation of antiapoptotic proteins (e.g., Bcl‐2 and Bcl‐XL) and the upregulation of proapoptotic proteins (e.g., Bax and Bcl‐XS). [67]. For As, human neuroblastoma cells treatment with As trioxide resulted in cell cycle arrest at the G2/M phase [68]. As increased cellular ROS levels and disrupted MMP, leading to the activation of caspase cascade reaction [69]. Cd induced apoptosis by increasing the Bax/Bcl‐2 ratio, while concurrently caused cell cycle arrest at the S and G2/M phases by upregulating the expression of p53, p21, and p27, and these combined effects led to apoptosis and cell cycle arrest in human astrocytes [70]. Hg exposure significantly reduced the expression of Cyclin D1 and Cyclin E, proteins crucial for the G1 and S phase transitions of the cell cycle, potentially leading to cell cycle arrest [71]. Additionally, Hg interfered with microtubule assembly and cellular energy metabolism by binding to sulfhydryl and selenol groups in proteins, thereby inducing apoptosis [72]. In vitro, study indicated that Pb nitrate (Pb(NO_3_)_2_) exhibited significant toxic effects on HL‐60 cells. Pb exposure was able to induce DNA damage, leading to cell cycle arrest at the G0/G1 phase and ultimately triggering apoptosis through the activation of caspase‐3 and the induction of nucleosome DNA fragmentation [73].

## 4 HMs, Mechanism, and Diseases

HMs are closely associated with a wide range of diseases, mainly affecting the nervous system, kidneys, cardiovascular system, reproductive and developmental system, and immune system, and they severely contribute to cancer risk. Of these, neurodegenerative diseases are particularly prominent as the nervous system is highly sensitive to HMs. Kidney damage and cardiovascular diseases are also major health concerns, while reproductive and developmental problems highlight the long‐term effects of HMs. The immune system is also significantly affected by HMs, which may lead to various immune‐related diseases. Also, certain HMs, such as Cr (VI) and Cd are generally recognized as carcinogens. These diseases are interrelated through OS, DNA damage, and cell signaling disruptions. Understanding these diseases and their mechanisms is critical to developing effective therapeutic strategies.

### 4.1 Nervous System

#### 4.1.1 Cognitive Dysfunction

Cognitive dysfunction, characterized by impairments in memory, attention, language, executive function, perception, and social skills. Numerous HMs, including Pb, Hg, and Cd, could induce OS in the brain. These metals could also generate ROS that overwhelm the body's antioxidant defense, causing oxidative damage to neurons and other brain cells. Additionally, normal cellular function could be disrupted by OS, leading to cognitive impairment [74, 75, 76]. In addition to OS, Cd exposure had been associated with the induction of inflammatory factors, such as TNF‐α, IL‐1β, and IL‐6, which contributed to cognitive dysfunction [77]. Hg exerted neurotoxic effects by hyperactivating N‐methyl‐D‐aspartate (NMDA) receptors, leading to cytoskeletal instability. The NMDA receptor, an ionotropic glutamate receptor, was involved in a variety of physiological processes, including synaptic plasticity, nervous system development, learning, and memory [78, 79]. HMs, including Pb, Cd, MeHg, and As, shared a common binding affinity with NMDA receptors, Na^+^–K^+^–ATPase pumps, biological Ca^2+^, and glutamate neurotransmitters, which might disrupt the balance between ROS and antioxidants in the hippocampal area, ultimately resulting in cognitive dysfunction [80].

#### 4.1.2 Neurodegenerative Diseases

Neurodegenerative diseases are characterized by a gradual and progressive loss of neuronal cell function and structure in specific brain regions, including Alzheimer's disease (AD), Parkinson's disease (PD), Amyotrophic lateral sclerosis (ALS), and Huntington's disease (HD) [81, 82]. Despite variations in clinical presentation and the specific brain regions affected, these diseases share some common pathological features: abnormal aggregation of proteins. In AD, amyloid beta (Aβ) protein plaques and neurofibrillary tangles (NFTs) serve as characteristic pathological hallmarks [83]. In PD, the aggregation of α‐synuclein (α‐syn) to form Lewy bodies is a defining pathological feature [84]. In ALS, the aberrant aggregation of TDP‐43 is associated with disease progression [85]. HD is associated with polyglutamine repeat expansion of Huntington protein [86]. Furthermore, mitochondrial dysfunction plays an important role in these neurodegenerative diseases, affecting energy metabolism and cell survival [87].

In AD, As exposure could increase the permeability of the blood–brain barrier, facilitating As accumulation in the brain and triggering neuroinflammation. Microglia, as the major immune cells in the central nervous system, are essential for neuroinflammatory responses and immune regulation [88, 89]. Furthermore, microglia activation was associated with impaired learning memory capacity in an As‐exposed mouse model [90, 91]. As exposure could also interfere with the processing of amyloid precursor protein (APP) to Aβ, a central characteristic of AD. Specifically, As elevated APP expression and facilitated the amyloidogenic pathway through activating β‐secretase and progerin [92]. Additionally, As exposure also affected mitochondrial dynamics, DNA repair pathways, and epigenetic alterations, all of which were potential mechanisms contributing to AD [93, 94].

Cd exposure might lead to neuronal death by triggering the mitochondrial apoptotic through multiple neurodegenerative signaling pathways. Research had demonstrated a significant correlation between blood Cd levels and AD mortality in the American elderly population, indicated that elevated blood Cd levels might serve as a significant predictor of increased AD mortality risk [95]. In addition, mice subjected to chronic Cd exhibited significant deterioration in memory and synaptic function. Furthermore, both subacute and chronic Cd exposure increased ROS production and suppressed antioxidant defense systems, including nuclear factor‐erythroid 2‐related factor 2 (Nrf2) and HO‐1. Also, Cd exposure activated the c‐Jun N‐terminal kinase 1 (JNK1) signaling pathway, which might further exacerbate the pathological changes associated with Aβ accumulation in AD [96]. Cd exposure influenced the phosphorylation state of tau protein, a critical step in the formation of NFTs. In AD, abnormal phosphorylation of tau proteins led to their detachment from microtubules, which in turn aggregated the formation of NFTs. Cd promoted the phosphorylation of tau protein through the activation of specific kinases, such as glycogen synthase kinase 3β (GSK‐3β) and cyclin‐dependent kinase 5 (CDK5) [97, 98]. Furthermore, even nontoxic concentrations of Cd could also promote the release of IL‐6 and IL‐8 by activating MAPK phosphorylation and NF‐κB signaling pathways [99].

Patients with AD exhibit elevated levels of Hg in the brain, blood, and tissue, with dental amalgam fillings being a significant exogenous source of brain Hg [100]. Aβ‐induced memory deficits in AD were manifested by Hg's detrimental effects on spatial learning and memory. Hg‐induced mitochondrial dysfunction further exacerbated spatial memory deficits in rats, as evidenced by increased ROS production, disruption of MMP, increased mitochondrial volume, GSH oxidation, and lipid peroxidation, which ultimately resulted in damage to the mitochondrial outer membrane. These alterations not only impaired hippocampal mitochondrial function, but also led to an elevated ADP/ATP ratio and decreased cytochrome *c* oxidase (complex IV) activity in the rat hippocampus [101].

Early exposure to Pb might cause young rats to exhibit addiction‐like symptoms of AD, with this exposure increasing the expression of APP and β‐secretase 1 (BACE1) in hippocampal and cortical regions, and further contributed to the accumulation of Aβ protein and the development of senile plaques in these regions [102]. Meanwhile, childhood Pb exposure promoted the expression of APP, BACE1, and transcription factor specific protein 1 (Sp1), which further facilitated Aβ deposition [103]. Furthermore, it had been shown that exposure to a mixture of As, Cd, and Pb induced early manifestations of AD‐like pathology in a synergistic manner, dependent on OS and inflammation [104].

In PD, the reduced expression of brain‐derived neurotrophic factor (BDNF) and phosphorylated GSK3β in the striatum of As‐exposed rats implied that dopamine (DA) signaling was affected here, as BDNF via its receptor tropomyosin receptor kinase B activated the PI3K/Akt signaling pathway, which subsequently inhibited GSK‐3β activity [105]. Additionally, decreased expression of pGSK3β might indicated increased GSK‐3β activity, which in turn led to higher expression of α‐Syn [106].

Cd exposure could trigger motor dysfunction, decrease the number of DA neurons, and cause neuropathological changes in midbrain regions.

Using untargeted lipidomic analyses, it had been demonstrated that Cd exposure led to alterations in the lipid composition of the midbrain, notably increasing levels of the proinflammatory sphingolipids, ceramide (Cer), SM, and ganglioside (GM3), and these lipid changes were associated with the development of neuroinflammation [107]. Cd could enter neuronal cells by influencing Zn transporter proteins, including ZIP6 and ZnT3. Mechanically, the upregulation of ZIP6 import proteins and downregulation of ZnT3 export proteins might lead to the accumulation of Cd in neurons, resulting in the accumulation of α‐syn protein and the disruption of neuroplasticity [108, 109].

As previously mentioned, Hg could be released from dental fillings and traversed the BBB to enter the brain, where it was also associated with PD strongly. Mechanically, Hg exhibited a variety of toxic effects, including the production of free radicals, autoimmune inflammation, and the attachment to sulfhydryl‐rich cell membranes in organelles, such as mitochondria, lysosomes, and the Golgi apparatus, all of which had been implicated in the pathogenesis of PD [110]. In addition, Hg had been found in neurons and oligodendrocytes, and usually colocalized with α‐syn [110]. This indicated that Hg was associated with the aggregation of α‐syn and might be involved in Lewy bodies formation.

Pb exposure had been shown to reduce dopaminergic neurotransmission through mitochondrial dysfunction, OS, and increased glial filaments in astrocyte. It readily crossed the BBB and was associated with alterations in various antioxidant enzymes and increased lipid peroxidation [111]. In addition, Pb could also induce OS via the activation of protein kinase C, leading to neurotoxicity [112]. Also, Pb caused hyperphosphorylation of Tau protein along with the accumulation of α‐Syn in the brain, leading to cellular apoptosis and autophagy activation, which contributed to the onset and progression of PD [113]. However, a study for HM detection of blood samples from PD patients found significantly lower Pb levels in PD patients compared with controls [114]. There are few studies on the correlation between Pb and PD, with inadequate sample sizes and a lack of comprehensive mechanistic studies, highlighting the need for further research in this area.

In ALS, Cd was able to penetrate neurons, leading to increased ROS within neurons and a concomitant reduction in their antioxidant defense mechanisms. Furthermore, Cd disrupted neuronal Ca^2+^ homeostasis, interfered with normal mitochondrial function, and activated cell death signaling pathways [115]. Cd exposure resulted in a significant increased expression level of *S100A2* gene, which encodes a highly specific regulatory Ca^2+^ binding protein [116]. Elevated intracellular Ca^2+^ level induced MAPK/mTOR activation, cytochrome oxidase subunit (COX‐I/II/III) dysfunction, MMP disruption, cleavage of caspase‐9 and caspase‐3, and OS‐induced elevated ROS levels [117, 118]. Besides calcium, Cd was able to displace Zn in the superoxide dismutase (SOD) enzyme, which caused SOD inactivity and subsequent OS [119].

TDP‐43, an RNA‐binding protein, plays a crucial role in ALS, and its aberrant aggregation is a major pathological feature of ALS [120]. MeHg was a powerful neurotoxin that caused neurotoxicity and neuronal cell death [121]. It promoted the aggregation of TDP‐43 in the nucleus and the formation of nuclear granules, while simultaneously reduced the availability of free TDP‐43 in the nucleus. In addition, MeHg disrupted TDP‐43 homeostasis in neurons, leading to increased transcriptional levels and enhanced splicing function [122]. Characteristics of MeHg‐induced ALS included oligodendrocyte damage, depletion of myelin basic protein (MBP), and degeneration of white matter, which in turn contributed to the demyelination and motor neuron death [123]. MeHg could also affect the Nrf2/HO‐1 signaling pathway, a potential target for neuroprotection in ALS. Nrf2 overexpression in astrocytes via the glial fibrillary acidic protein (GFAP) promoter reversed motor neuron damage in a mouse model of ALS [121, 124].

For familial‐ALS (f‐ALS), genes associated with f‐ALS include SOD1, transactivation response DNA binding protein, and angiogenin, with approximately 20% of f‐ALS associated with mutations in the Cu/Zn SOD1 gene [125, 126]. Pb weakened the SOD1 gene in rats, suggesting that Pb affected the normal folding process of the SOD1 protein, resulting in unfolded or misfolded SOD1 protein, which subsequently led to motor neuron apoptosis [127]. In addition, reduced activity of the mutant SOD1 protein and its impaired ability to scavenge superoxide radicals led to excessive production of H_2_O_2_, which triggered the aggregation of superoxide or hydroxyl radicals, and this might elucidate the mechanism by which the SOD1 mutation induced f‐ALS [128].

**FIGURE 2:**
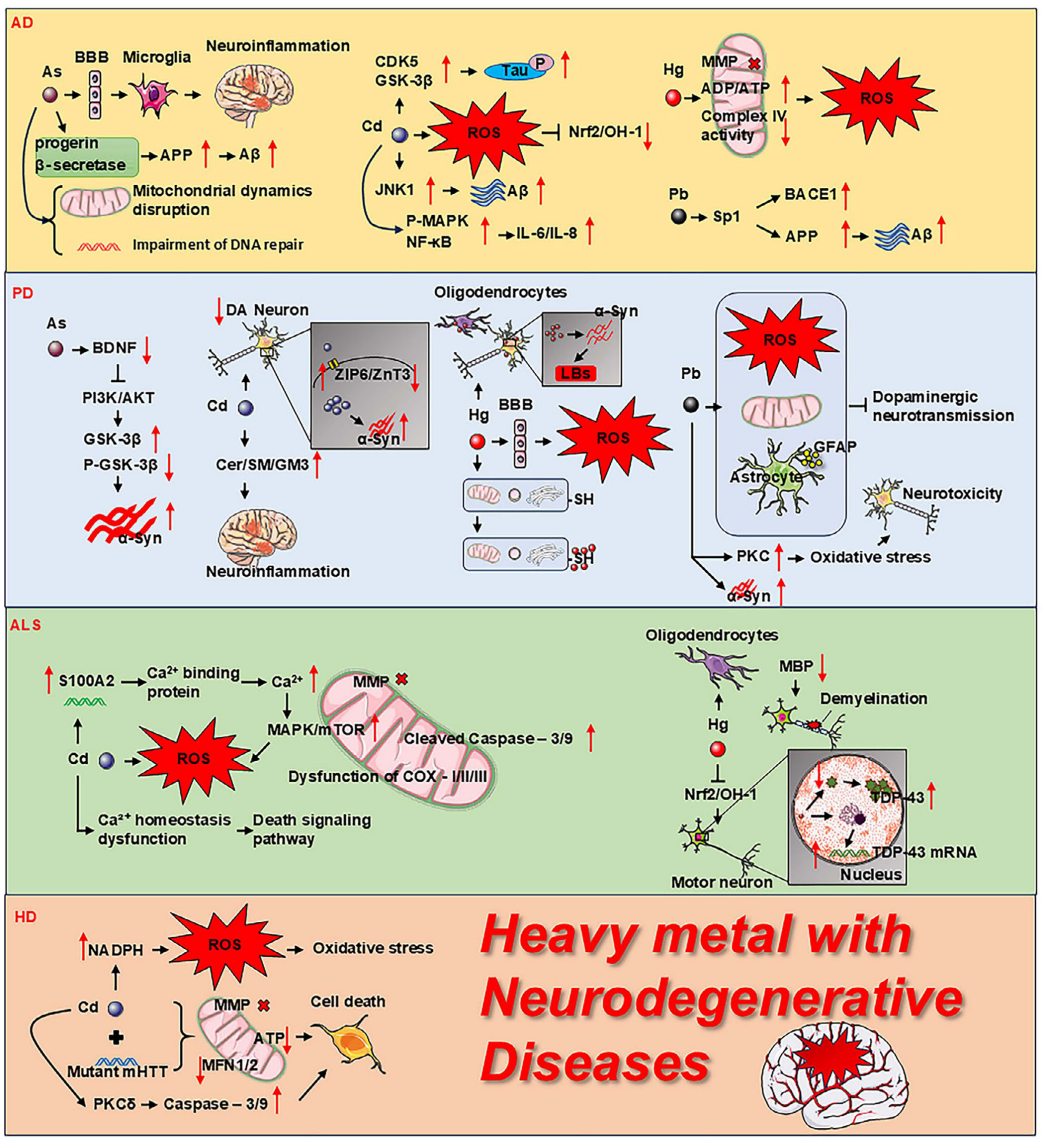
HMs affect the pathogenesis of neurodegenerative diseases through various pathways. AD: As increases Aβ production by affecting β‐secretase and APP expression, while also disrupting mitochondrial dynamics and DNA repair. As enters the brain through the BBB, and activates microglia, leading to neuroinflammation. Cd increases Aβ and IL‐6/IL‐8 production by activating the JNK1, P‐MAPK, and NF‐κB signaling pathways, resulting in neuroinflammation. Cd also affects CDK5, GSK‐3β, and Tau protein phosphorylation. Hg acts on mitochondria, causing the destruction of MMP, an increase in the ADP/ATP ratio, and a decrease in complex IV activity, ultimately leading to increased ROS levels. Pb increases the deposition of Aβ by affecting Sp1 expression, activating BACE1 and APP. PD: As reduces BDNF level, affecting the PI3K/AKT signaling pathway, thereby influencing GSK‐3β phosphorylation, which can lead to an increase in α‐syn. Cd affects the balance of zinc through ZIP6/ZnT3 transporters, thereby impacting the expression and aggregation of α‐syn. Additionally, Cd exacerbates neuroinflammation by increasing the levels of proteins related to neuroinflammation, such as Cer, SM, and GM3. Hg crosses the BBB and generates ROS in the brain. It can react with sulfhydryl groups in mitochondria, lysosomes, and the Golgi apparatus, leading to altered or inactivated functions. Hg entry into oligodendrocytes and neurons affect the aggregation of α‐Syn to form Lewy bodies. Pb affects mitochondria, induces ROS production, activates astrocytes, and thereby impeding dopaminergic neurotransmission. Pb activates PKC, increases OS, and promotes the aggregation of α‐Syn, leading to neurotoxicity in dopaminergic neurons. ALS: Cd disrupts calcium ion homeostasis, increases ROS production, activates the MAPK/mTOR signaling pathway, leading to mitochondrial dysfunction, and triggers the activation of apoptotic signaling pathways. Hg affects oligodendrocytes, reduces the level of MBP, causing demyelination, and inhibits the Nrf2/OH‐1 signaling pathway, thereby increasing the expression of TDP43 protein and mRNA in motor neurons. HD: Cd increases ROS production through NADPH oxidation, leading to OS. Additionally, mutated mHTT interacts with Cd to cause collapse of MMP, impairment of energy metabolism, and reduction of MFN1/2 levels. Meanwhile, Cd activates the PKCδ/Caspase‐3/9 signaling pathway, promoting cell death. α‐syn, α‐synuclein; Aβ, amyloid beta; AD, Alzheimer's disease; ADP/ATP, adenosine diphosphate/adenosine triphosphate; ALS, amyotrophic lateral sclerosis; APP, amyloid precursor protein; As, arsenic; BACE1, β‐secretase 1; BBB, blood–brain barrier; BDNF, brain‐derived neurotrophic factor; Cd, cadmium; CDK5, cyclin‐dependent kinase 5; Cer, ceramide; COX, cytochrome *c* oxidase; DA, dopamine; GFAP, glial fibrillary acidic protein; GM3, ganglioside; GSK‐3β, glycogen synthase kinase 3β; GSK‐3β, glycogen synthase kinase‐3β; HD, Huntington's disease; Hg, mercury; Hg, mercury; IL‐6/IL‐8, interleukin 6/interleukin 8; JNK1, c‐Jun N‐terminal kinase 1; LBs, Lewy body; MAPK, mitogen‐activated protein kinase; MBP, myelin basic protein; MFN1/2, mitofusin 1/2; mHTT, mutant huntingtin; MMP, mitochondrial membrane potential; mTOR, mechanistic target of rapamycin; NADPH, nicotinamide adenine dinucleotide phosphate; NF‐κB, nuclear factor kappa B; P‐GSK‐3β, phosphorylated glycogen synthase kinase‐3β; P‐MAPK, phosphorylated mitogen‐activated protein kinase; Pb, lead; PD, Parkinson's disease; PI3K/AKT, phosphoinositol‐3 kinase/protein kinase B; PKCδ, protein kinase c delta; PKC, protein kinase C; ROS, reactive oxygen species; S100A2, S100 calcium binding protein A2; SM, sphingomyelin; Sp1, specific protein 1; TDP‐43, TAR DNA binding protein 43; ZIP6, zinc finger protein 6; ZnT3, zinc transporter 3.

In HD, Cd accumulated predominantly in the striatum, and cells expressing mutant Huntington protein (mHTT) were more susceptible to cell death upon acute Cd exposure. This exposure synergistically interacted with mHTT to reduce MMP and ATP levels in striatal cells, and the expression of the profusion proteins MFN1 and MFN2 in mitochondria, leading to abnormalities in mitochondrial morphology and function that exacerbated cell death [129]. In addition, Cd activated NOX and produced more ROS, which in turn triggered OS. Notably, heterotrimeric HTT cells interacted with Cd exposure to activate protein kinase C δ (PKCδ), which activated caspase‐9 and caspase‐3‐mediated apoptosis, while simultaneously blocked the overexpression of extracellular signal regulated kinase (ERK) [130] (Figure 2).

### 4.2 Kidney Damage ‐ Renal Dysfunction

Renal dysfunction is characterized by impaired kidney function, resulting in the inability to effectively filter blood, excrete waste products, maintain electrolyte balance, and regulate blood pressure.

Cr (VI) is recognized as a toxic Cr in the environment. Clinically, exposure to Cr (VI) led to severe acute renal exhaustion, primarily affecting the epithelial cells of the proximal tubules. In the human immortalized proximal renal tubular epithelial cell line HK‐2, Cr (VI) treatment activated intrinsic and extrinsic apoptosis pathways, as evidenced by the increased expression of cleaved caspase‐8 and caspase‐3 [131].

As, predominantly excreted via the kidneys, could alter protein expression in renal tissues following exposure. Specifically, remarkable changes in the ETC, oxidative phosphorylation, mitochondrial structure, and apoptosis‐related protein expression were observed [132].

Cd accumulated mainly in the tubular cells of the kidney, and upon cell death, Cd was released and appeared in the urine. Within the proximal tubule, Cd was able to bind to molecules such as β2‐microglobulin, albumin, and lipid transport protein‐2, to form complexes. These complexes were internalized via the megalin and cubilin receptors located on proximal tubule cells, which were integral to the endocytosis pathway and responsible for intracellular uptake of Cd complexes [133, 134].

In systemic circulation, two transporter proteins, organic anion transporters 1 and 3 (OAT1 and OAT3), played an important role in the kidneys and are responsible for the transport of organic anions from blood into renal tubular cells. Hg entered the kidney cells via OAT1 and OAT3, where it undergone conversion from organic to inorganic forms by enzymatic or nonenzymatic processes [135, 136, 137]. Inorganic Hg deposition was strongly associated with ROS generation, metallothionein (MT) expression, cell apoptosis, and proximal tubule injury [138].

Pb‐induced pathological changes in the kidneys are mainly characterized by impaired proximal tubular function and might progress to Fanconi syndrome. Pb interacted with renal cell membranes and enzymes, disrupting energy metabolism, calcium homeostasis, glucose regulation, ion transport, and the renin‐angiotensin system [139].

### 4.3 Cardiovascular System

#### 4.3.1 Cardiovascular Disease

HMs, such as Cr, As, Cd, Hg, and Pb, are environmental pollutants prevalent in air, water, soil, food, and industrial products, and are associated with an elevated risk of cardiovascular disease [140]. As could cause cardiac arrhythmias, and it had been found that As_2_O_3_ could prolong the cardiac QT interval by inhibiting the processing of the human Ether‐à‐go‐go‐Related Gene protein to block rapid delayed rectifier potassium current (Ikr) in endoplasmic reticulum [141, 142]. Cd (VI) had a quenching effect on tryptophan and tyrosine residues in bovine hemoglobin, and this effect altered its secondary structure. In addition, Cd (VI) induced apoptosis and increased OS in vascular endothelial cells by modulating the p38 MAPK and JNK signaling pathways [143]. Myocardial cell electrophysiological disorder serve as crucial indicator of Cd‐induced myocardial cells injury. These disorders arise from the generation of stimulus electric potentials formed by ionic flow across cell membranes, involving ions include Na^+^, K^+^, and Ca^2+^. In biological systems, Cd existed as Cd^2+^ ions, which share structural similarities with Ca^2+^ ions [144]. Consequently, Cd^2+^ could compete with Ca^2+^ at the myocardial cell membrane, blocking its binding to calcium channel proteins and affecting Ca^2+^ influx, resulting in a decreased intracellular Ca^2+^ concentration and inhibited myocardial contractility [145]. Hg and its compounds damaged the vascular system primarily by triggering OS. Specifically, HgCl_2_ induced locally increased angiotensin II secretion, which might increase the activity of cyclooxygenase‐2 (COX‐2) and NOX, leading to ROS production and OS [146, 147, 148].There was an established association between Pb exposure and cardiovascular diseases, wherein chronic Pb exposure regulated NO signaling pathway. Then, NO activated soluble guanylate cyclase (sGC) to produce cyclic guanosine monophosphate (cGMP), which reduced Ca2+ concentration in vascular smooth muscle cells and promoted vasodilation. However, Pb exposure impaired this pathway by reducing sGC and cGMP, leading to decreased vasodilatory capacity [149].

#### 4.3.2 Effects of Cardiac Function

In a Drosophila model, cardiac direct exposure to Cd induced reversible cardiac arrest and disrupted calcium signaling [150]. Also, CdCl_2_ also reduced the expression and phosphorylation levels of the sarcoplasmic/endoplasmic reticulum calcium ATPase 2a (SERCA2a), adversely affecting the normal transport and utilization of calcium ions, ultimately leading to cardiac contractile dysfunction. This effect was more pronounced in males, whereas females did not exhibit similar changes, suggesting that sex differences might have an important role in Cd toxicity [151]. Early Pb exposure in mice led to offspring with reduced cardiac ejection fraction and increased left ventricular volume, accompanied by abnormal cardiomyocyte sarcomeres development, mitochondrial structural disorders, and impaired mitochondrial function [152]. Additionally, Pb exposure inhibited voltage‐gated calcium channels, impeded channel activation, reduced cardiac contractility, and increased the risk of arrhythmias [153].

### 4.4 Reproduction and Development

#### 4.4.1 Effects on the Reproductive System

Cr (VI) exposure increased the levels of OS markers, such as malondialdehyde (MDA) and protein carbonyls, while concurrently decreased the activities of antioxidant enzymes (e.g., SOD), in the ovaries of female rats, leading to ovarian dysfunction, adversely affecting follicular development and hormone synthesis [154]. Likewise, Cr (VI) induced OS damage in the male reproductive system by promoting free radical production, elevating MDA levels, and decreasing SOD and CAT activities, which resulted in reduced sperm count, lower viability, and tissue damage in reproductive organs [155]. Furthermore, As exposure during juvenile and puberty stages significantly affected reproductive development in female Sprague–Dawley rats by disrupting the estrous cycle, decreasing the number of primordial follicles and corpus luteum, increasing the number of atretic follicles, decreasing serum levels of estradiol, progesterone, and testosterone, elevating luteinizing hormone and follicle‐stimulating hormone levels, and selectively downregulating proteins associated with steroidogenesis, including follicle‐stimulating hormone receptor, steroidogenic acute regulatory protein, Cytochrome P450 family 17 subfamily A member 1 (CYP17A1), 3β‐hydroxysteroid dehydrogenase  (HSD3B1), and Cytochrome P450 family 19 subfamily A member 1 (CYP19A1) [156]. Cd disrupted spermatogenesis and compromised structural integrity of testicular tissue by inducing OS and DNA damage, and upregulated the expression of epidermal growth factor receptor (EGFR) and its downstream signaling pathways (e.g., p‐AKT, NF‐κB, COX‐2, etc.), which resulted in a decrease in spermatozoa viability and quantity [157]. HgCl_2_ exposure exerted significantly toxic to the male reproductive system, primarily through mechanisms involving the testicular immunosuppression and fibrosis, which were related to the inhibition of the CD74 signaling pathway [158]. Pb caused testicular damage, decreased sperm quality, and induced reproductive dysfunction by promoting OS, disrupting mitochondrial function in sperm, decreasing sperm motility, and downregulating steroidogenesis‐related enzymes [159].

#### 4.4.2 Effects on Embryo Development

Cr (VI), exemplified by potassium dichromate, showed significant toxicity to mouse embryos cultured in vitro, mainly by inhibiting blastocyst formation, and inducing OS and apoptosis in embryonic cells [160]. As caused delayed preimplantation embryonic development by inducing redox imbalance, characterized by decreased GSH levels and increased p66Shc levels, and interfered with extracellular amino acid metabolism in the embryo [161]. Additionally, maternal Cd exposure impaired preimplantation embryonic development in mice by inducing OS, interfering with epigenetic modifications, such as elevated levels of HDAC 1 and altered DNA methylation of the *H19* gene, and increasing DNA damage, ultimately leading to embryonic death and developmental arrest [162].

### 4.5 Cancer Risk

#### 4.5.1 Carcinogenicity of HMs

A recognized association exists between HMs and carcinogenicity, the process by which normal cells are transformed into cancerous cells. HMs might contribute to the cancer development through a variety of mechanisms. They could damage DNA and induce genetic mutations, which might lead to cancer. Additionally, HMs also produced ROS and triggered chronic inflammation, which in turn led to OS and DNA damage, further promoting carcinogenesis [163]. In addition, HMs could also alter gene expression patterns through epigenetic changes, such as DNA methylation and histone modifications, leading to abnormal gene expression that derived cancer progression. Moreover, HMs also interfered with signaling pathways involved in cell growth, differentiation, and apoptosis, and disruption of these pathways might promote uncontrolled cell proliferation and evasion of programmed death, both of which are hallmarks of cancer [164].

#### 4.5.2 Associations with Specific Cancer Types

**FIGURE 3:**
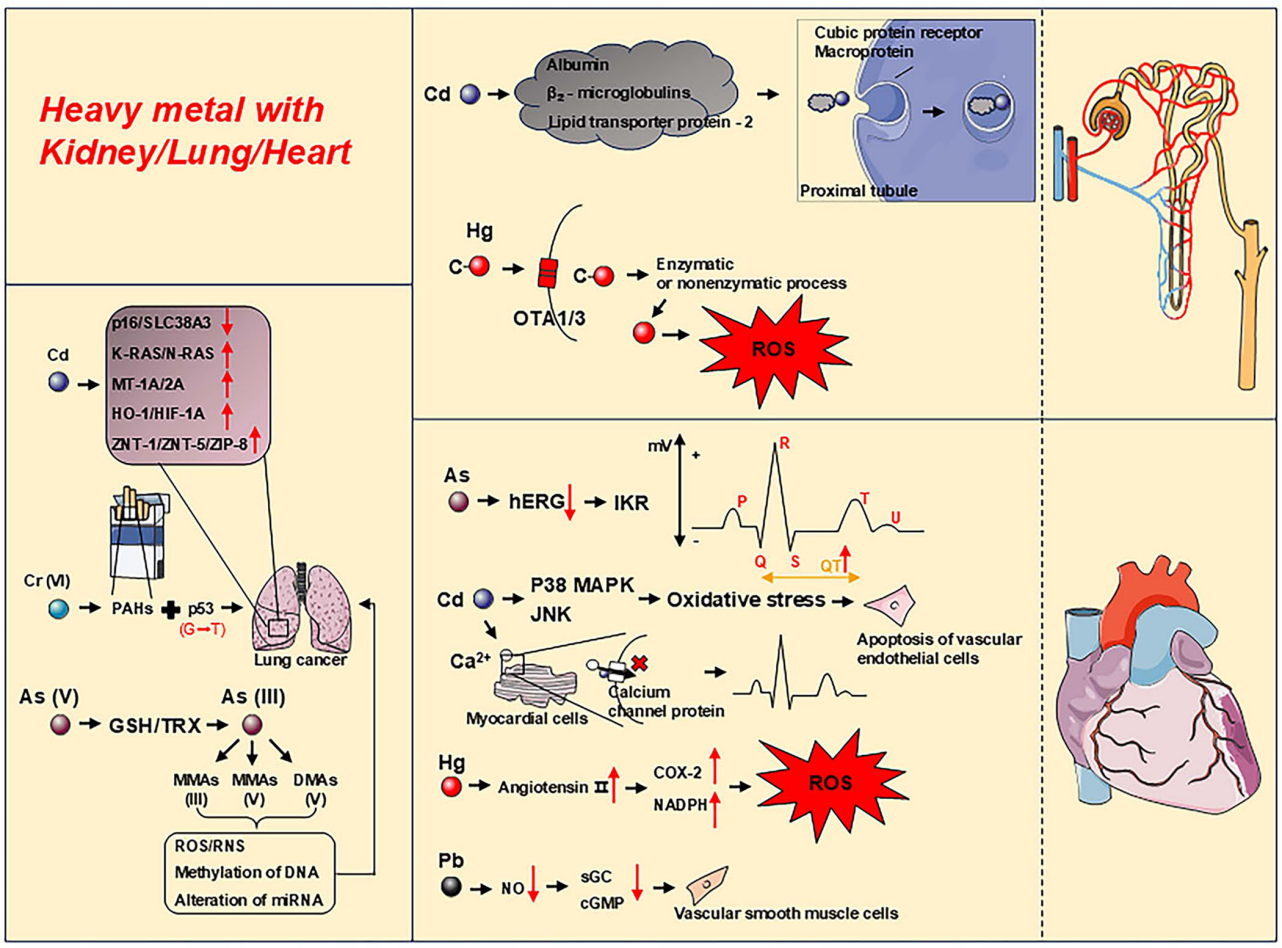
Mechanisms of HMs in the kidney, heart, and cancer. *Kidney*: Cd binds with albumin, β2‐microglobulin, and lipid transport protein‐2 and is endocytosed via megalin and cubilin receptors on the proximal tubular cells, thereby interfering with the normal metabolic processes of the kidney. Organic Hg interacts with OAT1/3 and is converted into inorganic Hg through enzymatic or nonenzymatic processes, leading to ROS production. *Heart*: As can prolong the QT interval of the heart. Cd induces vascular endothelial cell apoptosis by activating P38 MAPK or JNK signaling pathway, and competing with Ca^2+^ to inhibit the contractility of myocardium cells. Hg increases the secretion of angiotensin II and causes ROS generation. Pb impairs the NO signaling pathway and reduces the vasodilatory capacity of blood vessels. *Lung*: Cd regulates the expression of multiple signaling pathways and promotes the occurrence of lung cancer. Cr (VI) promotes the binding of PAHs in cigarettes to the p53 gene in the lung and also increases the mutation of guanine to thymine in the p53 gene. As (V) is reduced to As (III) in the presence of GSH and TRX, which is further metabolized into carcinogens such as MMA (III), MMA (V) and DMA (V), causing lung cancer. As (V), arsenic (V); Cd, cadmium; cGMP, cyclic guanosine monophosphate; COX‐2, cyclooxygenase 2; Cr, chromium; DMA, dimethylarsinate; GSH, glutathione; GSH/TRX, glutathione/thioredoxin; hERG, human ether‐à‐go‐go‐related gene; Hg, mercury; HIF‐1A, hypoxia‐inducible factor‐1A; HO‐1, heme oxygenase‐1; IKR, inward rectifier potassium current; JNK, c‐Jun N‐terminal kinase; MMA, monomethylarsonate; MT‐1A/2A, metallothionein‐1A/2A; MT, metallothionein; NADPH, nicotinamide adenine dinucleotide phosphate; NO, nitric oxide; P38 MAPK, P38 mitogen‐activated protein kinase; p53, tumor protein 53; PAHs, polycyclic aromatic hydrocarbons; ROS/RNS, reactive oxygen species/reactive nitrogen species; sGC, soluble guanylate cyclase; ZNT‐1/ZNT‐5/ZIP‐8, zinc transporter‐1/5/Zinc induced protein‐8.

Cr (VI) is a prevalent environmental and industrial pollutant that is strongly associated with an increased incidence of lung cancer. Polycyclic aromatic hydrocarbons (PAHs) from cigarette smoking are the main carcinogens responsible for lung cancer. Cr (VI) exposure had a high proportion of guanine to thymine (G to T) mutations in the *p53* gene, which is a hallmark mutation pattern observed in PAHs. In addition, Cr (VI) facilitated the binding of PAHs to the p53 gene in the lungs [165]. Similarly, As exhibited high toxicity and pathogenicity, especially in the lungs [166]. Upon As entered the human body, it undergone a series of reduction, oxidation, and methylation reactions, to transform into carcinogenic substances. Specifically, As (V) was reduced to As (III) in the presence of GSH and thioredoxin (TRX). Subsequently, As (III) was further methylated during in vivo metabolism to produce more carcinogenic As metabolites: trivalent monomethylarsenic acids MMAs (III), pentavalent MMAs (V), and pentavalent dimethylarsenic acids DMAs (V). Alterations triggered by these processes at the genetic and epigenetic levels included the generation of ROS and reactive nitrogen species, modifications in DNA methylation patterns, alterations in miRNA expression, and variations in histone modifications [167]. Chronic Cd exposure in human lung cells in vitro had been shown to exhibit increased invasiveness, colony formation, cell proliferation, downregulation of tumor suppressor genes p16 and SLC38A3, upregulation of oncoproteins K‐RAS and N‐RAS, increased in MT‐1A and MT‐2A, and activation of OS adaptive response genes HO‐1 and HIF‐1A, as well as increased metal transporter genes ZNT‐1, ZNT‐5 and ZIP‐8 expression [168] (Figure 3).

Cd is a known environmental carcinogen that is strongly associated with the development of breast cancer. Cd, an endocrine disruptor, could promote breast cancer cell proliferation and survival by activating the interaction between estrogen receptor alpha (ERα) and proto‐oncogene c‐jun (c‐Jun) [169]. Alternatively, Cd could also promote breast cancer cell proliferation by binding to the membrane estrogen receptor GPR30 and activating the downstream Erk‐1/2 signaling pathway [170]. Similarly, Pb belongs to a class of metalloestrogens that could activate ERα, thereby triggering estrogen‐like biological effects that promoted cell proliferation and the development of estrogen‐dependent breast cancer. Furthermore, both Cd and Pb affected DNA repair mechanisms, causing DNA damage and increased genetic instability [171]. Interestingly, Cr (II) was able to activate ERα, whereas Cr (III) cannot [172]. For As, it was shown that individuals who are adept at metabolizing inorganic As to (MMA (III)), but less adept at further metabolizing MMA (III) to dimethylarsenic acid (DMA (III)), were at higher breast cancer risk [173]. The impact of Hg on breast cancer breast cancer has been relatively understudied; however, evidence suggests its potential carcinogenic properties. Hg was capable of damaging DNA, and mutations in genes associated with DNA repair (e.g., BRCA1) had been linked to breast cancer development [174, 175].

For other types of cancer, Cd exposure induced malignant cell transformation in prostate epithelial cells mainly by increasing the expression level of the antiapoptotic gene Bcl‐2, which in turn blocked the JNK signaling pathway, and ultimately results in a significant increase in the antiapoptotic capacity of prostate epithelial cells, leading to prostate cancer development [176]. Similarly, the prostate is a target organ for the carcinogenic effects of inorganic As [177]. Chronic As exposure led to the malignant transformation of prostate epithelial cells, manifested by genomic DNA hypomethylation and K‐ras overexpression [178]. In addition, long‐term low‐dose Cd exposure promoted the invasive and metastatic ability of colorectal cancer cells through the unique activation of the EGFR signaling pathway, which is evidenced by persistent EGFR signaling and activation of Akt/mTOR cascade, whereas blocking EGFR eliminated the promotion of Cd on the metastasis of colorectal cancer cells to the liver [179]. Chronic Cr(VI) exposure was found to exacerbate colorectal cancer induced by 1,2‐dimethylhydrazine, with an underlying mechanism involving Cr (VI) induced OS and macrogenomic analyses revealing alterations in gut microbiota composition, with downregulation of the abundance of *Firmicutes* and *Bacteroidetes*, remarkable increased abundance of *Verrucomicrobia*, and decreased levels of certain short‐chain fatty acids generating bacteria [180].

### 4.6 Immune System

HMs can damage immune cells and influence immune function. In allergic contact dermatitis, Cr (VI) penetrated the skin barrier, was reduced to Cr(III), and subsequently formed immunogenic complexes with skin proteins, which activated antigen‐presenting cells, subsequently triggering a cascade of immune responses involving effector T cells, regulatory T cells, and Tγδ cells, ultimately leading to allergic reactions [181]. Cr exposure could also disrupt the balance of Th1/Th2/Th17 cytokines and regulate the humoral immune response, resulting in the disruption of immune homeostasis, and causing inflammation as well as other adverse outcomes [182]. As exposure had a wide range of toxic effects on the immune system, including the inhibition of T‐cell activation, the alteration in a variety of cytokines (IL‐2, IL‐4, IL‐5, IL‐10, IFN‐γ, and TNF‐α expressions, the effects on leukocyte function and adhesion, and impairment of both humoral and cellular immune responses, which could lead to an increased risk of infection and inflammatory diseases [183]. For Cd, immune cells were both targets of Cd toxicity and effectors of Cd‐induced immune responses and toxic effects [184]. In primary T lymphocytes derived from BALB/c mice, Cd induced immunotoxicity by selectively acting on CD4+ and CD8+ T cells. Of these, CD4+ T cells were more sensitive to Cd‐induced OS and apoptosis, and Cd significantly inhibited Th1 and Th2 cytokines expression in a dose‐dependent manner [185]. Under the context of Hg‐induced inflammation and autoimmune responses, Hg activated innate immune cells (neutrophils and macrophages) and induced the activation of adaptive immune cells (T and B cells), to trigger autoimmune diseases [186]. Among workers with chronic exposed to Pb in battery factories, decreased helper T‐lymphocyte numbers and immunoglobulins (IgG and IgM), and complement proteins (C3 and C4), suggested that Pb exhibited an inhibitory effect on the immune system, thus weakening the body's immune response [187].

## 5 Therapeutic Intervention

Addressing the toxic effects of HMs requires a comprehensive combination of therapeutic strategies covering chelation therapy, antioxidants and free radical scavengers, supportive care, preventive measures, and emerging therapeutic technologies. Chelation therapy promotes the elimination of HMs from the body through the formation of complexes between chelating agents and HMs. Antioxidants and free radical scavengers are effective in attenuating OS and ROS damage and protecting cells from further damage. Supportive therapy, including nutritional support and symptomatic treatment, can help improve patient prognosis and relieve symptoms. Preventive measures are taken at the source to reduce HMs exposure and reduce health risks through environmental interventions and personal protection. Notably, emerging therapeutic strategies such as nanotechnology, gene therapy, and microbial modulation offer new directions and ideas for the treatment of HMs toxicity and show broad application prospects. Together, these strategies provide comprehensive protection against HMs toxicity.

### 5.1 Chelation Therapy

#### 5.1.1 Types and Mechanisms of Chelating Agents

HMs, such as Cr, As, Cd, Hg, and Pb, pose significant threats to human health. Chelating agents played an important role in detoxifying these metals, and they are categorized into two main types: mercapto and nonmercapto chelating agents. Mercapto chelators, including agents like bimercaptopropanol (BAL), dimercaptosuccinic acid (DMSA), dimercaptopropanesulfonic acid (DMPS), Monoisoamyl DMSA (MiADMSA), penicillamine (PEN), and lipoic acid (LA), contain sulfhydryl groups that enable them to form stable chelates with HMs [188, 189]. In contrast, nonmercapto chelators, primarily represented by ethylenediaminetetraacetic acid (EDTA), do not contain sulfhydryl groups, but possess multiple carboxyl groups that facilitate chelation with HM ions [190, 191].Chelating mechanism of the aforementioned agents mainly involve: (1) Ligand bond formation, wherein the chelating agent interact with HM ions through its active functional groups, such as sulfhydryl and carboxylic acid group, to form ligand bonds and generate stable chelates, thereby reducing the bioavailability of HMs [192, 193]. (2) Enhanced excretion, as the chelates formed with HMs are usually readily excreted, facilitating the removal of HMs from the body [194].

#### 5.1.2 Clinical Application of Chelation Therapy

The earliest HM chelators were EDTA and BAL, and their clinical use has been limited by the inconvenience of administering, inherent toxicity, and the potential for increased neurotoxicity of certain metals [195]. Notably, DMPS and DMSA, representing a new generation of chelating agents, offered superior safety and therapeutic benefit over BAL in the treatment of As and Hg toxicity, as these agents exhibited a higher therapeutic index, and did not result in the redistribution of toxic metals to the brain [196, 197]. Specifically, BAL, also known as British Anti‐Lewis Aerosol, is to detoxify from As or Hg. Upon interaction with Lewis gas, BAL is able to produce stable and nontoxic As compounds [198]. In the decades following Second World War, BAL was widely used in the detoxification of HMs poisoning with inorganic Hg, As, Sb, gold (Au), and bismuth (Bi) [199]. However, high doses of BAL caused a range of serious adverse effects, including vasoconstriction of small arteries, increased blood pressure, headaches, blurred vision, and numbness in the hands [200]. Due to its high toxicity, nowadays, BAL is only used for a short period of time when the patient's life is threatened or in an emergency situation where is experiencing acute As poisoning. In addition, given its narrow therapeutic range and its propensity to redistribute toxic elements to the brain, BAL had been replaced in most cases by DMSA or DMPS [195]. DMSA, a disulfide compound and an analog of BAL, can specifically bind to Pb, reducing its absorption and retention in the gastrointestinal tract, and subsequently lowering blood Pb concentrations. However, short‐term administration of DMSA, it is easy to free Pb from bone and redistribute it, causing rebound elevation of blood Pb [201]. The hydrophilic properties of DMSA enable considerable absorption in the gastrointestinal tract, thus the oral administration pathway gives it a significant advantage over BAL [202]. Furthermore, DMSA had a large therapeutic window and the lowest toxicity among dithiols [203]. DMPS, another analog of BAL, is not considered an appropriate treatment for Pb poisoning [204]. Whereas, DMPS had shown remarkable efficacy in the field of detoxification of Hg poisoning and had become a critical therapeutic agent for this condition, its effectiveness in the treatment of Pb and As poisoning was limited [205, 206, 207]. MiADMSA is a potential candidate drug that is still in the developmental stage. It had been shown to be effective in experimental animal models of acute and chronic Pb and As poisoning, exerting antioxidant properties, promoting MT synthesis, and also reversing Cd‐induced oxidative damage [208, 209]. Additionally, MiADMSA had no significant effect on maternal and fetal developmental toxicity, which was higher than DMSA but lower than BAL [210, 211]. In summary, the field of chelation therapy has progressed significantly from the early use of EDTA and BAL to the development of safer and more effective chelating agents such as DMSA and DMPS. As research progresses, emerging drug candidates such as MiADMSA offer new hope for improving the treatment of HMs toxicity.

#### 5.1.3 Preclinical Animal Experiments and Clinical Trials

Preclinical experiments serve as a fundamental approach to elucidate the mechanisms underlying the toxicity of HMs and to develop potential therapeutic interventions. By simulating human HMs exposure scenarios in animal models, researchers are able to gain an in‐depth understanding of the effects of HMs on different organ systems and evaluate the efficacy and safety of therapeutic strategies. For example, in high‐dose Pb‐exposed mice, intraperitoneal injection of DMSA significantly reduced Pb levels in the kidney, bone, and brain, which was superior to other well‐known chelating agents such as calcium sodium ethylenediaminetetraacetate (CaNa_2_EDTA), Zn sodium ethylenediaminetetraacetate, and the trisodium salt of Zn dimercaptopropionic acid [212]. Additional studies had found that in Pb‐exposed rats, the combination of intraperitoneal DMSA and CaNa_2_EDTA was more effective at removing Pb from organs and bones than either drug alone or in combination with other drugs (e.g., DMPS) without causing the redistribution of Pb to other organs [213]. In a study on the prevention of As poisoning in rats, the combination of intraperitoneal DMSA and MiADMSA performed excellently, significantly reducing As‐induced OS and effectively removing As from the blood and liver [214]. In addition, after As exposure in rats, N‐acetylcysteine (NAC) in combination with DMSA significantly reduced OS and removed As from organs [215]. Also, As exposure elevated OS, increased ROS and MDA, and inhibited cholinesterase activity in Swiss white rat brains. Intraperitoneal injection of vitamin E and coenzyme Q10 attenuated oxidative damage, restored cholinesterase activity, and improved brain function by scavenging free radicals and modulating SOD and GSH‐Px activities [216]. In a study of Cd‐induced kidney toxicity in mice, the combination of intraperitoneal injection of DMSA and subcutaneous injection of Zn diethylenetriaminepentaacetate trisodium (ZnDTPA) was more effective than individual use [217]. Notably, certain chelating agents may pose potential risks in the treatment of HMs poisoning. For example, BAL increased the uptake of Hg into the brain tissue of mice, leading to the redistribution of Hg in the brain [218]. Apart from synthetic chelating agents, natural products also showed potential for HMs management. Aqueous extracts of onion and garlic prevented Cd‐induced OS injury in rat kidneys, in addition, garlic extracts reduced Pb concentrations in rat liver, kidney, brain, and bone [219, 220]. Chelation of Pb by oral administration of an alcoholic extract of cilantro significantly reduced Pb deposition in mouse femurs, decreased renal injury, and decreased urinary excretion of δ‐aminolevulinic acid, showing preventive effects against Pb poisoning [221].

HMs chelators have also demonstrated promising therapeutic effects in clinical trials. A prospective experimental research had shown that sequestration treatment with long‐term, intermittent intravenous CaNa_2_EDTA significantly reduced accumulated Pb levels in healthy asymptomatic individuals [222]. Moreover, in a case study involving 17 Pb‐poisoned individuals, intravenous DMSA treatment significantly increased urinary Pb excretion and rapidly relieved neurologic and gastrointestinal symptoms associated with Pb poisoning [223]. In clinical trials conducted in a Au‐mining region of the Philippines, 106 patients with Hg poisoning were treated with 400 mg per day of oral DMPS. Although blood Hg levels did not significantly decrease, urinary Hg excretion increased up to 85‐fold [224]. Currently, the combined treatment strategy for HMs poisoning is still in the exploratory stage in clinical trials. Future research could explore the combined use of antioxidants, vitamins, and chelating agents, based on preclinical animal studies, to evaluate their collective efficacy (Table 1).

**TABLE 1:** Animal experiments and clinical trials on the therapy of different heavy metal poisoning.

HMs	Experimental subject	Treatment	Drug delivery pathway	Consequence	References
Pb	Mouse	DMSA	Intraperitoneal injection	Reduced Pb levels in kidneys, bone, and brain	Jones et al. [212]
Pb	Rat	DMSA + CaNa_2_EDTA	Intraperitoneal injection	Reduced Pb levels in bone and brain	Tandon et al. [213]
As	Rat	DMSA + MiADMSA	Intraperitoneal injection	Reduced oxidative stress and cleared As from blood and liver	Flora et al. [214]
As	Rat	DMSA + NAC	Intraperitoneal injection	Reduced oxidative stress and removed As from organs	Flora et al. [215]
As	Swiss white rat	Vitamin E and coenzyme Q10	Intraperitoneal injection	Reduced oxidative damage and restored cholinesterase activity	Sharma et al. [216]
Cd	Mouse	DMSA + ZnDTPA	Intraperitoneal injection; hypodermic injection	Combination of drugs to alleviate renal toxicity	Eybl et al. [217]
Hg	Mouse	BAL	Intraperitoneal injection	Increased Hg uptake in brain tissue, redistribution of Hg in the brain	Berlin et al. [218]
Cd	Rat	Onion and garlic water extract	Oral	Alleviate renal oxidative stress	Suru et al. [219]
Pb	Rat	Garlic extract	Oral	Reduced Pb concentration in the liver, kidney, brain, and bone	Senapati et al. [220]
Pb	Mouse	Cilantro alcoholic extract	Oral	Reduced Pb deposition in femur, ameliorated Pb‐induced kidney damage	Aga et al. [221]
Pb	Healthy, asymptomatic individuals	CaNa_2_EDTA	Intraperitoneal injection	Reduced accumulated Pb levels in the body	Petteruti et al. [222]
Pb	Pb‐poisoned individuals	DMSA	Oral	Increased urinary lead excretion and relieved neurological and gastrointestinal symptoms	Bradberry et al. [223]
Hg	Patients in the gold‐mining area	DMPS	Oral	Significantly increased urinary Hg excretion	Drasch et al. [224]

Abbreviations: As, arsenic; BAL, bimercaptopropanol; CaNa_2_EDTA, calcium sodium ethylenediaminetetraacetate; Cd, cadmium; DMPS, dimercaptopropanesulfonic acid; DMSA, dimercaptosuccinic acid; Hg, mercury; MiADMSA, monoisoamyl DMSA; NAC, N‐acetylcysteine; Pb, lead; ZnDTPA, zinc diethylenetriaminepentaacetate trisodium.

### 5.2 Antioxidant and Free Radical Scavenger

#### 5.2.1 The Role of Antioxidants

Antioxidants play a crucial role in mitigating HM toxicity, and they alleviate the oxidative injury and toxic effects through multiple mechanisms. (1) Antioxidants directly neutralized free radicals and prevented them from attacking cell membranes, proteins, and DNA, thereby protecting cells from OS [225]. (2) LA antioxidants were able to form stable complexes with HM ions, reducing their bioavailability and toxicity in the body [226]. (3) Antioxidants activated and regenerated endogenous antioxidants, such as GSH, vitamins C and E, strengthening cellular antioxidant defense mechanisms [227, 228]. (4) Antioxidants mitigated HM‐induced mitochondrial damage, protected mitochondrial function, and reduced ROS generation [229].

#### 5.2.2 Application of Free Radical Scavenger

Clinically, the main free radical scavengers used included a variety of natural and synthetic compounds that act by neutralizing free radicals, reducing OS, and safeguarding cells from damage. These agents were employed not only in the prevention and treatment of diseases associated with OS, such as cardiovascular diseases, cancer, and neurodegenerative diseases, but also played an important role in delaying the aging process and improving overall health. The following are some of the free radical scavengers that are widely used in clinical practice: (1) *Vitamin C*: a water‐soluble antioxidant that collaborated with vitamin E to protect lipids from peroxidation [230]. (2) *GSH*: the predominant intracellular antioxidants involved in a variety of biosynthetic and metabolic processes [231]. (3) *NAC*: an antioxidant containing sulfhydryl groups, which acted as a precursor to GSH, to enhance the antioxidant capacity of cells through multiple mechanisms [232]. (4) Melatonin, the hormone secreted by the pineal gland, was integral to regulate the sleep–wake cycle, and is also widely acknowledged for its powerful antioxidant properties [233]. (5) *Natural flavonoids*: such as curcumin and resveratrol, had antioxidant activity [234].

### 5.3 Supportive Treatment

#### 5.3.1 Nutritional Support

Nutritional therapy for HM intervention was a comprehensive strategy used that encompassed multiple aspects. Prior to initiating HM chelation therapy, it is imperative to ensure adequate supplementation of nutrients required for liver detoxification, including essential fatty acids, phospholipids, antioxidants, vitamins, and minerals. Dietary adjustments should be made to minimize the intake of foods that caused allergies or intolerances, while prioritizing clean, organic foods to reduce the sources of toxicity. Propolis extracts and proanthocyanidins might serve as alternative dietary supplements for mitigating Pb and Cd exposure in the body [235]. Intake of specific phytonutrients, including lignans, glucosides, catechins, isoflavones, and ellagic acid, was recommended to enhance liver detoxification mechanisms. Also, increasing the intake of probiotic‐rich foods available in the market, usually found in dairy products such as cheese and yoghurt, as well as certain cereals could be beneficial. Specific probiotics, such as Lactobacillus, was able to scavenge HMs, thereby reducing their detrimental effects by either forming complexes with them or altering their bioavailability [236, 237, 238, 239]. Collectively, these strategies constitute an integrated nutritional therapy plan aimed at alleviating the advance effects of HMs on human health.

#### 5.3.2 Symptomatic Treatment

Symptomatic treatment of HM poisoning involved a series of therapeutic interventions targeting various symptoms and indications caused by HM toxicity, to alleviate clinical symptoms, prevent the progression of the diseases, protect the function of vital organs, and promote patient recovery. For HM through oral intake, emetic and stomach lavage should be performed promptly to eliminate the toxic residues in the stomach. However, for patients experiencing severely poisoning or for whom stomach lavage was ineffective, blood purification techniques such as haemodialysis or peritoneal dialysis should be considered to remove the HM ions from the blood. For symptoms such as nausea and vomiting, antiemetic drugs might be administered, while antispasmodic drugs were appropriate for managing symptoms such as abdominal pain and diarrhea.

### 5.4 Prevention and Reduction of Exposure

#### 5.4.1 Environmental Interventions

Environmental interventions for HMs include the following specific strategies. Implementing stringent industrial emission standards to curtail the release of HMs from factories and mines [240]. Adoption of advanced waste treatment and recycling technologies to mitigate the risk of HMs entering the environment [241]. Establishment of an environmental monitoring system to regularly assess the levels of HMs in soil, water, and air, for timely detection and intervention [242]. Employment of chemical reagents to transfer HMs from the soil to an aqueous solution, called drenching, to reduce the soil HM content [243]. Using techniques such as phytoextraction, phytovolatilization, phytofiltration, and microbial restoration, to diminish HMs through biological processes in soil and water [244, 245].

#### 5.4.2 Personal Protection and Health Education

Personal protection measures against HMs are multifaceted, covering various aspects from workplace safety and daily habits to diet and health defense. It was important to wear protective gear, including gloves, lab coats, goggles, long trousers, and closed shoes, when handling HMs. These strategies are instrumental in mitigating skin exposure and inhalation of HMs. When performing HM‐related experiments, it is essential to cover laboratory bench with protective mats to prevent any spills or drips, which should subsequently be disposed of as hazardous waste. Moving away from areas with high levels of air pollution and industrial emissions can significantly reduce HM exposure. Air purifiers are advisable when living in urban areas with poor air quality. Sweating is the body's natural way of eliminating toxins, including HMs. Participation in activities such as exercise, saunas, and hot yoga can eliminate toxins through perspiration. Cigarette smoke contains detrimental chemicals, including HMs like Cd and Pb. Thus, quitting smoking and avoiding second‐hand smoke are crucial for significantly reduce HM exposure. Raise public awareness of the hazards of HMs through educational activities to inform individuals about HMs sources, their effects on health, and preventive measures. Strengthen legislation and enforcement of HM pollution, establish a robust policy and regulatory framework, delineating responsibilities, authorities, and regulatory bodies, increase penalties for sources of pollution, and promote the fulfilment of environmental protection obligations by enterprises and individuals.

### 5.5 Emerging Therapeutic Strategies

Previously, the article mainly discussed the existing treatment strategies for HMs poisoning, including chelation therapy, antioxidants, and free radical scavengers, as well as supportive care and preventive measures. Although these methods can alleviate the symptoms of HMs poisoning at a certain extent, with the development of science and technology and the in‐depth understanding of HMs toxicity, have highlighted the limitations of the traditional treatments. Consequently, there is an urgent need to develop new therapeutic approaches to effectively address the health risks associated with HM poisoning. Here, this section will focus on emerging therapeutic strategies that are expected to provide new directions and ideas for the treatment of HMs poisoning.

#### 5.5.1 Nanotechnology

Nanotechnology has great potential in the treatment of HMs toxicity, where nanoparticles can be engineered to specifically bind and remove HMs from the body [246, 247]. Nanoparticles are characterized by small size, large specific surface area, high surface activity, and could efficiently adsorb and transport HMs. Silicon dioxide nanoparticles were able to adsorb metal ions efficiently due to their large specific surface area and suitable adsorption sites, thus reducing the binding and toxic effects of HMs on biomolecules [248]. In addition, nanomaterials could also form stable complexes with HMs through chemical reactions, facilitating their elimination [249]. Moreover, by surface functionalization (e.g., introduction of carboxyl, amino, or sulfhydryl groups), the adsorption capacity of nanomaterials for HMs (such as Pb, Cd, and Hg) could be significantly enhanced [250]. More importantly, nanotechnology could also achieve targeted therapy by combining nanoparticles with ligands, such as monoclonal antibodies, folic acid, or peptides, to specifically target cells or tissues, thereby improving the therapeutic efficacy [251]. Nanoparticles exhibit significant potential for the adsorption and therapeutic management of HMs. However, challenges persist regarding their biosafety, stability, and how to realize efficient and specific HMs adsorption, necessitating further in‐depth investigation.

#### 5.5.2 Gene Therapy

HMs exposure leads to abnormal gene expression or mutations in cells, which can lead to diseases. Clustered regularly interspaced short palindromic repeats associated protein 9 (CRISPR/Cas9), a breakthrough gene‐editing technology, is emerging as a highly promising tool for gene therapy [252]. Using this technology, it is possible to repair or replace damaged genes and restore normal gene functions [253]. Gene therapy technology regulates gene metabolism, facilitates the detoxification of HMs, and enhances tolerance to HMs [254]. However, the application of these technologies need be executed with stringent precision and safety controls, to avoid potential off‐target effects [255].

#### 5.5.3 Microbiome Regulation

Microbes play a unique and important role in the management of HMs pollution by transforming these metals into less toxic forms through complex biochemical processes, such as oxidation, reduction, methylation, and demethylation. For example, some microbes are able to oxidize the highly toxic As (III) to the less toxic As (V), or reduce the strongly oxidizing Cr (VI) to the less toxic Cr (III), thus effectively reducing the harmful effects of these HMs on the environment and living organisms [256, 257]. In addition, microorganisms could convert HMs into volatile compounds through enzymatic reactions, further reducing their toxicity and bioavailability in soil and water. For instance, the enzyme mercuric reductase (MerA) was able to reduce Hg (II) to volatile monomeric Hg (0), allowing it to escape from the environment, and thus reducing its accumulation in ecosystems. Likewise, As methyltransferases could convert As (V) to volatile methylarsenic compounds that are relatively less toxic and easier to remove from the environment [258, 259]. Interestingly, microorganisms exhibited a unique capacity for HMs adsorption and accumulation, characterized by their cell wall components and intracellular metal‐binding proteins (e.g., MT and phytochelatins) that could efficiently adsorb and accumulate HMs [260]. Besides, extracellular polysaccharides secreted by microorganisms also had significant HMs adsorption capacity [261] (Table 2).

**TABLE 2:** Therapeutic intervention strategies of HMs on human diseases.

Therapeutic strategy	Measures	Mechanism of action	Applicable heavy metals	Advantages/disadvantages	Research progress	References
Supportive therapy	Blood purification (dialysis/plasma exchange)	Directly removes free HMs from the blood	Acute Hg, Pb poisoning	A: fast‐acting; D: equipment dependent and costly	For severe cases	/
	Nutritional support (propolis extract, proanthocyanidins, Lactobacillus)	Forms complexes with HMs	Cd, Pb	A: preventative; D: long‐term supplementation required	Supported by epidemiological evidence	Halttunen et al. [238]
Protective measure	Environmental remediation (phytoextraction/chemical drenching)	Soil purification using super‐enriched plants	Cd, As, Pb in soil	A: source control; D: long lead times	Higher costs in promotion	Zhang et al. and Ojha et al. [243, 244]
	Personal protection (masks/gloves)	Reduce occupational exposure (e.g., mining, battery manufacturing)	All heavy metals	A: simple and effective; D: dependent on adherence	Statutorily mandated	/
Emerging technology	Nanomaterials (SiO_2_)	Adsorption of HMs with high specific surface area or targeted delivery of chelating agents	Pb, Cd, Hg	A: efficient targeting; D: potential biotoxicity	Laboratory stage, safety validation required	Olawade et al. and Xia et al. [250, 251] Samani et al. [248]
	Gene therapy (CRISPR–Cas9)	Repair of HMs‐induced mutations (e.g. p53) or enhancement of detoxification gene expression	As, Cr (VI)	A: potential for eradication; D: technological immaturity, ethical controversy	Basic research phase	Ma et al. [252]
	Microbial remediation (MerA genetically engineered bacteria)	Microbial reduction of Hg^2^⁺ to Hg⁰ volatilization, or degradation of organic arsenic	Hg, As	A: environmentally friendly; D: limited environmental adaptability	Some of the engineered bacteria have been field tested	Hemmat‐Jou et al and Naguib et al. [258, 259]

Abbreviations: As, arsenic; Cd, cadmium; Cr, chromium; CRISPR–Cas9, clustered regularly interspaced short palindromic repeats associated protein 9; Hg, mercury; Pb, lead; SiO_2_, silicon dioxide.

## 6 Conclusion And Prospect

### 6.1 Summary of HM Toxicity

HMs exert detrimental effects on organisms through various mechanisms inducing OS, triggering neuroinflammation, interfering with protein homeostasis, and disrupting organelle function. HMs can interact with biological macromolecules such as proteins, enzymes, and nucleic acids, in cells, resulting in their structure and function damage, which consequently affects the normal cellular physiological activities. For example, HMs could enhance ROS production, compromise cellular antioxidant defense, trigger lipid peroxidation, oxidative modification of proteins, and DNA damage, ultimately leading to cellular dysfunction and death. HMs might also interfere with cell signaling pathways, affect the cell cycle and apoptosis processes, as well as disrupt the normal functions of mitochondria and endoplasmic reticulum, further exacerbating cellular damage.

The toxic effects of HMs are particularly pronounced in the neurological system, where they can inflict severe damage to nerve cells, contributing to the development of neurodegenerative diseases. HMs can traverse the BBB, then interfering with the nerve cell functions, destroying cellular structure, affecting the signaling pathway, and triggering pathological protein aggregation, neuroinflammation and increased OS. Over time, the accumulation of HM damage to the nervous system led to progressive degradation of neuronal function and structural integrity. This long‐term cumulative effect eventually precipitated a spectrum of neurodegenerative diseases, including AD, PD, ALS, and HD. These diseases usually manifest with a complex set of symptoms that seriously affect patient's quality of living. *AD*: the main manifestations are memory loss, delayed thinking, and cognitive decline, which may eventually lead to complete loss of self‐care. *PD*: the predominantly movement disorders, such as tremors, muscle rigidity, and bradykinesia, accompanied by cognitive decline. *ALS*: primarily affects the motor neurons, leading to muscle weakness and atrophy, which may eventually affect respiratory function. *HD*: presents with involuntary dance‐like movements, cognitive deficits, and abnormal mental behaviors, accompanied by depression and anxiety. These symptoms not only cause great disturbance to the daily lives of the patients, but also seriously affect their self‐care, social activities and work ability, which brings a substantial burden on family and society.

The health effects of HMs are multisystemic, long term, and cumulative. These hazards are not limited to a single organ, but may affect multiple physiological systems through intricate biological mechanisms. Specifically, HM damage to the kidneys might lead to metabolic disorders, which in turn affected the cardiovascular system. Toxicity to the reproductive system might affect overall health through endocrine disruption. In addition, the carcinogenic mechanism of HMs involved DNA damage, OS, and immunosuppression at various levels, rendering their impacts on health even more complex and far‐reaching. The effects of HMs on the kidneys, the cardiovascular system, the reproductive system, and cancer, are multifaceted and have long‐term cumulative consequences, requiring enhanced environmental management and public health interventions to reduce the risks to human health caused by HMs.

### 6.2 Implications for Public Health and Environmental Protection

HMs exert a profound and critical impact on public health and environmental protection. From the environmental protection perspective, HMs pollution is detrimental in numerous ways. Within water systems, industrial wastewater emissions, mining, and other events cause large quantities of HMs such as Hg, Cd, and Pb into rivers, lake, and ocean. These not only alter the chemical properties of water and affect the habitat of aquatic organisms, leading to the death or mutation of fish and other aquatic organisms, but also enter the food chain through bioaccumulate in aquatic organisms, posing potential threats to human health. In soil, the use of HM‐containing pesticides and fertilizers in agriculture and the accumulation of industrial waste residues have caused the HMs concentrations exceeding permissible limits, further destroying the soil structure, reducing soil fertility, affecting nutrients and water absorption by plant roots, and resulting in a reduction in crops yields and quality. Additionally, these HMs may also accumulate in plants and further enter the human body through food consumption. In the atmosphere, metal refining, vehicle exhaust emissions, etc. will release Pb, Hg and other HMs. They can remain suspended in the air, to reduce air quality, and can enter the human body via breathing. Long‐term deposition of these HMs in the lung organs can damage to the respiratory system.

From a public health perspective, the threat posed by HMs to human health warrants serious attention. The neurological system is particularly susceptible to the detrimental effects of HMs. Specifically, Pb could affect the intellectual development of children, leading to cognitive decline, learning disabilities, and other problems, and will also lead to neurasthenia and memory loss in adults; Hg could damage to the brain and nervous system, triggering vision loss, limb coordination disorders, and so on. The immune system was also compromised by HMs. For instance, Cd might interfere with the normal function of the immune system, making people more susceptible to infectious diseases, and reducing the body's resistance. Furthermore, HMs had an adverse effect on the reproductive system. Both Pb and Hg could pass through the placental and blood‐testis barriers, and affect the formation and development of reproductive cells, which might lead to infertility and fetal abnormalities, posing a serious threat to human reproduction and offspring health.

Collectively, to protect public health and the environment, it is imperative to implement effective strategies to deal with HMs. On the one hand, it is necessary to strengthen environmental supervision, strictly control the emission of HMs from industrial enterprises, promote cleaner production technologies, and minimize the generation and release of HMs in the manufacturing processes. On the other hand, it is essential to elevate the public awareness regarding the hazards of HMs, advocate environmentally sustainable lifestyles, reduce the use and disposal of HMs‐containing products, and ensure proper garbage classification and recycling. Implementing these comprehensive strategies will effectively mitigate the threat to the environment and human health posed by HMs pollution and to foster sustainable development.

## Author Contributions

All authors contributed to the writing of the manuscript and the design of the figures and tables. All the authors participated in the design of the figures and tables. F.Z., Y.F.C., Y.J.Z., and C.C. contributed to the writing of the manuscript. All authors have read and approved the final manuscript

## Conflicts of Interest

The authors declare no conflicts of interest.

## Ethics Statement

The authors have nothing to report.

## Data Availability

The authors have nothing to report.

## References

[1] F. Dragan , M. Lestyan , V. V. Lupu , et al., “The Threat of Mercury Poisoning by Fish Consumption,” Applied Sciences 13 (2023): 369.

[2] L. Cilliers and F. Retief , “Chapter 14‐Lead Poisoning and the Downfall of Rome: Reality or Myth?” Toxicology in Antiquity (Second Edition) (2019), 221–229.

[3] J. McCurry , “Japan remembers Minamata,” Lancet 9505 (2006): 99–100. 10.1016/S0140-6736(06)67944-0 16419257

[4] X. Zhou , Q. Wang , and X. Yang , “[Progresses on mechanisms of pharmacological and toxicological effects of cinnabar],” Zhongguo Zhong Yao Za Zhi = Zhongguo Zhongyao Zazhi = China Journal of Chinese Materia Medica 34, no. 22 (2009): 2843–2847. 20209942

[5] C. J. Chen , S. K. Wu , Y. B. Wang , J. F. Hou , L. Ma , and X. Y. Sun , “[Recent Researches of Synthetic mercury Sulfide in Traditional Medicine System],” Zhongguo Zhong Yao Za Zhi = Zhongguo Zhongyao Zazhi = China Journal of Chinese Materia Medica 37, no. 19 (2012): 2968–2970. 23270244

[6] Y. Zhao , B. Yuan , K. Onda , et al., “Anticancer Efficacies of Arsenic Disulfide Through Apoptosis Induction, Cell Cycle Arrest, and Pro‐Survival Signal Inhibition in Human Breast Cancer Cells,” American Journal of Cancer Research 8, no. 3 (2018): 366–386. 29636995 PMC5883090

[7] L. Wei , R. Xue , P. Zhang , Y. Wu , X. Li , and F. Pei , “(1)H NMR‐Based Metabolomics and Neurotoxicity Study of Cerebrum and Cerebellum in Rats Treated With Cinnabar, a Traditional Chinese Medicine,” Omics : a Journal of Integrative Biology 19 (2015): 490–498. 26110755 10.1089/omi.2015.0042

[8] Z. Xiang , S. Wu , L. Zhu , K. Yang , D. Lin , “Pollution Characteristics and Source Apportionment of Heavy Metal(loid)s in Soil and Groundwater of a Retired Industrial Park,” Journal of Environmental Sciences 143 (2024): 23–34. 10.1016/j.jes.2023.07.015 38644020

[9] D. Zhao , P. Wang , and F. J. Zhao , “Toxic Metals and Metalloids in Food: Current Status, Health Risks, and Mitigation Strategies,” Current Environmental Health Reports 11 (2024): 468–483. 39352604 10.1007/s40572-024-00462-7 PMC11588791

[10] H. Ali and E. Khan , “What are Heavy Metals? Long‐Standing Controversy Over the Scientific use of the Term ‘Heavy Metals’ – Proposal of a Comprehensive Definition,” Toxicological & Environmental Chemistry 100 (2018): 6–19.

[11] K. Jomova , S. Y. Alomar , E. Nepovimova , K. Kuca , and M. Valko , “Heavy Metals: Toxicity and Human Health Effects,” Archives of Toxicology 99 (2025): 153–209. 39567405 10.1007/s00204-024-03903-2 PMC11742009

[12] T. J. van der Kuijp , L. Huang , and C. R. Cherry , “Health Hazards of China's Lead‐Acid Battery Industry: A Review of its Market Drivers, Production Processes, and Health Impacts,” Environmental Health : a Global Access Science Source 12 (2013): 61. 23915167 10.1186/1476-069X-12-61 PMC3750646

[13] A. Rashid , B. J. Schutte , A. Ulery , et al., “Heavy Metal Contamination in Agricultural Soil: Environmental Pollutants Affecting Crop Health,” Agronomy 13 (2023): 1521.

[14] M. Adnan , B. Xiao , M. U. Ali , et al., “Heavy Metals Pollution From Smelting Activities: A Threat to Soil and Groundwater,” Ecotoxicology and Environmental Safety 274 (2024): 116189. 38461579 10.1016/j.ecoenv.2024.116189

[15] X. Ma , Z. Sha , Y. Li , et al., “Temporal‐Spatial Characteristics and Sources of Heavy Metals in Bulk Deposition Across China,” The Science of the Total Environment 926 (2024): 171903. 38527555 10.1016/j.scitotenv.2024.171903

[16] C. Li , X. Zhang , M. Dong , X. Han , “Progress on Crowding Effect in Cell‐Like Structures,” Membranes 12 (2022): 593. 35736300 10.3390/membranes12060593 PMC9228500

[17] C. Wang , P. Zou , C. Yang , et al., “Dynamic Modifications of biomacromolecules: Mechanism and Chemical Interventions,” Science China Life Sciences 62 (2019): 1459–1471. 31555961 10.1007/s11427-019-9823-1

[18] A. VonHandorf , H. A. Zablon , and A. Puga , “Hexavalent Chromium Disrupts Chromatin Architecture,” Seminars in Cancer Biology 76 (2021): 54–60. 34274487 10.1016/j.semcancer.2021.07.009 PMC8627925

[19] S.‐G. Park and D. Butcher , “Investigation of the Interaction Between Arsenic Species and Thiols via Electrospray Ionization Tandem Mass Spectrometry,” Microchemical Journal 95 (2010): 57–66.

[20] S. Shila , M. Subathra , and M. A. Devi , C. Panneerselvam , “Arsenic Intoxication‐Induced Reduction of Glutathione Level and of the Activity of Related Enzymes in rat Brain Regions: Reversal by DL‐Alpha‐Lipoic Acid,” Archives of Toxicology 79 (2005): 140–146. 15798887 10.1007/s00204-004-0614-8

[21] C. A. Vergara‐Gerónimo , A. León Del Río , M. Rodríguez‐Dorantes , P. Ostrosky‐Wegman , and A. M. Salazar , “Arsenic‐Protein Interactions as a Mechanism of Arsenic Toxicity,” Toxicology and Applied Pharmacology 431 (2021): 115738. 34619159 10.1016/j.taap.2021.115738

[22] S. S. Katti and T. I. Igumenova , “Protein‐Cadmium Interactions in Crowded Biomolecular Environments Probed by In‐cell and Lysate NMR Spectroscopy,” Biorxiv (2024): 2023.11.03.565546

[23] S. A. Rupa , M. A. M. Patwary , M. M. Matin , W. E. Ghann , J. Uddin , and M. Kazi , “Interaction of Mercury Species With Proteins: Towards Possible Mechanism of Mercurial Toxicology,” Toxicology Research 12 (2023): 355–368. 37397928 10.1093/toxres/tfad039 PMC10311172

[24] M. J. Warren , J. B. Cooper , S. P. Wood , and P. M. Shoolingin‐Jordan , “Lead Poisoning, Haem Synthesis and 5‐Aminolaevulinic Acid Dehydratase,” Trends in Biochemical Sciences 23 (1998): 217–221. 9644976 10.1016/s0968-0004(98)01219-5

[25] T. L. DesMarais and M. Costa , “Mechanisms of Chromium‐Induced Toxicity,” Current Opinion in Toxicology 14 (2019): 1–7. 31511838 10.1016/j.cotox.2019.05.003 PMC6737927

[26] Y. Hu , J. Li , B. Lou , et al., “The Role of Reactive Oxygen Species in Arsenic Toxicity,” Biomolecules 10 (2020): 240. 32033297 10.3390/biom10020240 PMC7072296

[27] F. Qu and W. Zheng , “Cadmium Exposure: Mechanisms and Pathways of Toxicity and Implications for Human Health,” Toxics 12 (2024): 388. 38922068 10.3390/toxics12060388 PMC11209188

[28] J. Albrecht , E. Matyja , “Glutamate: A Potential Mediator of Inorganic Mercury Neurotoxicity,” Metabolic Brain Disease 11 (1996): 175–184. 8776719 10.1007/BF02069504

[29] V. Matović , A. Buha , D. Ðukić‐Ćosić , and Z. Bulat , “Insight Into the Oxidative Stress Induced by Lead and/or Cadmium in Blood, Liver and Kidneys,” Food and Chemical Toxicology 78 (2015): 130–140. 25681546 10.1016/j.fct.2015.02.011

[30] A. Samborska , Z. Stepniewska , and W. Stepniewski , “Influence of Different Oxidation States of Chromium (VI, III) on Soil Urease Activity,” Geoderma 122 (2004): 317–322.

[31] S. Ran , X. Gao , M. Ma , et al., “NaAsO2 Decreases GSH Synthesis by Inhibiting GCLC and Induces Apoptosis Through Hela cell Mitochondrial Damage, Mediating the Activation of the NF‐κB/miR‐21 Signaling Pathway,” Ecotoxicology and Environmental Safety 234 (2022): 113380. 35298964 10.1016/j.ecoenv.2022.113380

[32] O. I. Oluranti , E. A. Agboola , N. E. Fubara , M. O. Ajayi , and O. S. Michael , “Cadmium Exposure Induces Cardiac Glucometabolic Dysregulation and Lipid Accumulation Independent of Pyruvate Dehydrogenase Activity,” Annals of Medicine 53 (2021): 1108–1117. 34259114 10.1080/07853890.2021.1947519 PMC8280890

[33] B. Fernandes Azevedo , L. Barros Furieri , F. M. Peçanha , et al., “Toxic Effects of Mercury on the Cardiovascular and Central Nervous Systems,” Journal of Biomedicine & Biotechnology 2012 (2012): 949048. 22811600 10.1155/2012/949048 PMC3395437

[34] C. Wang , H. Shang , S. Zhang , et al., “Hexavalent Chromium Disrupts the Skin Barrier by Targeting ROS‐Mediated Mitochondrial Pathway Apoptosis in Keratinocytes,” Chemico‐Biological Interactions 379 (2023): 110523. 37146930 10.1016/j.cbi.2023.110523

[35] W. Guo , D. Li , Y. Zhai , et al., “Differential Interaction Modes of As(III)/As(V) With Microbial cell Membrane Induces Opposite Effects on Organic Contaminant Biodegradation in Groundwater,” Environment International 193 (2024): 109074. 39426033 10.1016/j.envint.2024.109074

[36] E. Van Kerkhove , V. Pennemans , and Q. Swennen , “Cadmium and Transport of Ions and Substances Across cell Membranes and Epithelia,” Biometals 23 (2010): 823–855. 20582616 10.1007/s10534-010-9357-6

[37] R. Notariale , E. Längst , P. Perrone , D. Crettaz , M. Prudent , and C. Manna , “Effect of Mercury on Membrane Proteins, Anionic Transport and Cell Morphology in Human Erythrocytes,” Cellular Physiology and Biochemistry 56 (2022): 500–513. 36126286 10.33594/000000572

[38] M. B. Dlamini , Z. Gao , Hasenbilige , et al., “The Crosstalk Between Mitochondrial Dysfunction and Endoplasmic Reticulum Stress Promoted ATF4‐Mediated Mitophagy Induced by Hexavalent Chromium,” Environmental Toxicology 36 (2021): 1162–1172. 33650752 10.1002/tox.23115

[39] C. Prakash and V. Kumar , “Arsenic‐Induced Mitochondrial Oxidative Damage is Mediated by Decreased PGC‐1α Expression and its Downstream Targets in rat Brain,” Chemico‐Biological Interactions 256 (2016): 228–235. 27425645 10.1016/j.cbi.2016.07.017

[40] J. J. V. Branca , A. Pacini , M. Gulisano , N. Taddei , C. Fiorillo , and M. Becatti , “Cadmium‐Induced Cytotoxicity: Effects on Mitochondrial Electron Transport Chain,” Frontiers in Cell and Developmental Biology 8 (2020): 604377. 33330504 10.3389/fcell.2020.604377 PMC7734342

[41] J. Y. Lee , G. W. Hwang , A. Naganuma , and M. Satoh , “Methylmercury Toxic Mechanism Related to Protein Degradation and Chemokine Transcription,” Environmental Health and Preventive Medicine 25 (2020): 30. 32680455 10.1186/s12199-020-00868-3 PMC7469908

[42] M. Chlubek and I. Baranowska‐Bosiacka , “Selected Functions and Disorders of Mitochondrial Metabolism Under Lead Exposure,” Cells 13 (2024): 1182. 39056765 10.3390/cells13141182 PMC11275214

[43] Y. Ma , S. Li , S. Tang , et al., “Clusterin Protects Against Cr(VI)‐Induced Oxidative Stress‐Associated Hepatotoxicity by Mediating the Akt‐Keap1‐Nrf2 Signaling Pathway,” Environmental Science and Pollution Research International 29 (2022): 52289–52301. 35257348 10.1007/s11356-022-19118-w

[44] C. C. Lin , C. S. Pan , C. Y. Wang , S. W. Liu , L. D. Hsiao , and C. M. Yang , “Tumor Necrosis Factor‐Alpha Induces VCAM‐1‐Mediated Inflammation via c‐Src‐Dependent Transactivation of EGF Receptors in Human Cardiac Fibroblasts,” Journal of Biomedical Science 22 (2015): 53. 26173590 10.1186/s12929-015-0165-8 PMC4502472

[45] C. Y. Zhang , A. J. Ou , L. Jin , et al., “Cadmium Exposure Triggers Alveolar Epithelial cell Pyroptosis by Inducing Mitochondrial Oxidative Stress and Activating the cGAS‐STING Pathway,” Cell Communication and Signaling 22 (2024): 566. 39587603 10.1186/s12964-024-01946-7 PMC11590492

[46] S. H. Kim and R. P. Sharma , “Mercury‐Induced Apoptosis and Necrosis in Murine Macrophages: Role of Calcium‐Induced Reactive Oxygen Species and p38 Mitogen‐Activated Protein Kinase Signaling,” Toxicology and Applied Pharmacology 196 (2004): 47–57. 15050407 10.1016/j.taap.2003.11.020

[47] J. Guo , R. Li , Z. Ouyang , et al., “Insights Into the Mechanism of Transcription Factors in Pb2+‐Induced Apoptosis,” Toxicology 503 (2024): 153760. 38387706 10.1016/j.tox.2024.153760

[48] Z. Fang , M. Zhao , H. Zhen , L. Chen , P. Shi , and Z. Huang , “Genotoxicity of Tri‐ and Hexavalent Chromium Compounds In Vivo and Their Modes of Action on DNA Damage In Vitro,” PLoS ONE 9 (2014): e103194. 25111056 10.1371/journal.pone.0103194 PMC4128586

[49] X. Zhou , R. M. Speer , L. Volk , L. G. Hudson , and K. J. Liu , “Arsenic Co‐Carcinogenesis: Inhibition of DNA Repair and Interaction With Zinc Finger Proteins,” Seminars in Cancer Biology 76 (2021): 86–98. 33984503 10.1016/j.semcancer.2021.05.009 PMC8578584

[50] M. Filipic , T. Fatur , and M. Vudrag , “Molecular Mechanisms of Cadmium Induced Mutagenicity,” Human & Experimental Toxicology 25 (2006): 67–77. 16539211 10.1191/0960327106ht590oa

[51] L. H. Wyatt , A. L. Luz , X. Cao , et al., “Effects of Methyl and Inorganic Mercury Exposure on Genome Homeostasis and Mitochondrial Function in Caenorhabditis Elegans,” Dna Repair 52 (2017): 31–48. 28242054 10.1016/j.dnarep.2017.02.005 PMC5394729

[52] S. Hemmaphan and N. K. Bordeerat , “Genotoxic Effects of Lead and Their Impact on the Expression of DNA Repair Genes,” International Journal of Environmental Research and Public Health 19 (2022): 4307. 35409986 10.3390/ijerph19074307 PMC8998702

[53] Z. Wang , Z. Liu , P.‐S. Wang , et al., “Epigenetic Downregulation of O6‐Methylguanine‐DNA Methyltransferase Contributes to Chronic Hexavalent Chromium Exposure‐Caused Genotoxic Effect and Cell Transformation,” Environmental Pollution 341 (2024): 122978. 37995958 10.1016/j.envpol.2023.122978 PMC11372728

[54] M. Iyer , U. Anand , S. Thiruvenkataswamy , et al., “A Review of Chromium (Cr) Epigenetic Toxicity and Health Hazards,” The Science of the Total Environment 882 (2023): 163483. 37075992 10.1016/j.scitotenv.2023.163483

[55] Y. Qin , H. Xu , Y. Xi , et al., “Effects of the SEMA4B Gene on Hexavalent Chromium [Cr(VI)]‐Induced Malignant Transformation of Human Bronchial Epithelial Cells,” Toxicology Research 13 (2024): tfae030. 38464415 10.1093/toxres/tfae030 PMC10919774

[56] T. F. Cheng , S. Choudhuri , and K. Muldoon‐Jacobs , “Epigenetic Targets of Some Toxicologically Relevant Metals: A Review of the Literature,” Journal of Applied Toxicology 32 (2012): 643–653. 22334439 10.1002/jat.2717

[57] Z. Xing and B. P. Tu , “Mechanisms and Rationales of SAM Homeostasis,” Trends in Biochemical Sciences 50 (2025): 242–254. 39818457 10.1016/j.tibs.2024.12.009 PMC11890959

[58] H. Li , L. Wu , F. Ye , et al., “As3MT via Consuming SAM is Involved in Arsenic‐Induced Nonalcoholic Fatty Liver Disease by Blocking m6A‐Mediated miR‐142‐5p Maturation,” The Science of the Total Environment 892 (2023): 164746. 37301390 10.1016/j.scitotenv.2023.164746

[59] N. F. Fitz , A. Barchowsky , R. Koldamova , and I. Lefterov , “Genome‐Wide Alteration of Histone Methylation Profiles Associated With Cognitive Changes in Response to Developmental Arsenic Exposure in Mice,” Toxicology Reports 9 (2022): 393–403. 35299870 10.1016/j.toxrep.2022.03.008 PMC8920871

[60] S. Hu , A. Song , L. Peng , et al., “H3K4me2/3 Modulate the Stability of RNA Polymerase II Pausing,” Cell Research 33 (2023): 403–406. 36922644 10.1038/s41422-023-00794-3 PMC10156655

[61] V. Hure , F. Piron‐Prunier , T. Yehouessi , et al., “Alternative Silencing States of Transposable Elements in Arabidopsis Associated With H3K27me3,” Genome biology 26 (2025): 11. 39833858 10.1186/s13059-024-03466-6 PMC11745025

[62] S. Frings , R. Schmidt‐Schippers , and W.‐K. Lee , “Epigenetic Alterations in Bioaccumulators of Cadmium: Lessons From Mammalian Kidneys and Plants,” Environment International 191 (2024): 109000. 39278047 10.1016/j.envint.2024.109000

[63] A. H. Guo , S. Kumar , and D. B. Lombard , “Epigenetic Mechanisms of Cadmium‐Induced Nephrotoxicity,” Current Opinion in Toxicology 32 (2022): 100372. 37193357 10.1016/j.cotox.2022.100372 PMC10168606

[64] T. Ke , A. A. Tinkov , A. V. Skalny , et al., “Epigenetics and Methylmercury‐Induced Neurotoxicity, Evidence From Experimental Studies,” Toxics 11 (2023): 72. 36668798 10.3390/toxics11010072 PMC9860901

[65] G. Bjørklund , L. Pivina , M. Dadar , Y. Semenova , S. Chirumbolo , and J. Aaseth , “Mercury Exposure, Epigenetic Alterations and Brain Tumorigenesis: A Possible Relationship?,” Current Medicinal Chemistry 27 (2020): 6596–6610. 31566127 10.2174/0929867326666190930150159

[66] M. Luo , Y. Xu , R. Cai , et al., “Epigenetic Histone Modification Regulates Developmental Lead Exposure Induced Hyperactivity in Rats,” Toxicology Letters 225 (2014): 78–85. 24291742 10.1016/j.toxlet.2013.11.025

[67] S. Ceryak , C. Zingariello , T. O'Brien , and S. R. Patierno , “Induction of Pro‐Apoptotic and Cell Cycle‐Inhibiting Genes in Chromium (VI)‐Treated Human Lung Fibroblasts: Lack of Effect of ERK,” Molecular and Cellular Biochemistry 255 (2004): 139–149. 14971655 10.1023/b:mcbi.0000007270.82431.3e

[68] X. Xiong , Y. Li , L. Liu , et al., “Arsenic Trioxide Induces Cell Cycle Arrest and Affects Trk Receptor Expression in Human Neuroblastoma SK‐N‐SH Cells,” Biological Research 51 (2018): 18. 29898774 10.1186/s40659-018-0167-6 PMC5998579

[69] V. Selvaraj , M. Y. Armistead , M. Cohenford , and E. Murray , “Arsenic Trioxide (As(2)O(3)) Induces Apoptosis and Necrosis Mediated Cell Death Through Mitochondrial Membrane Potential Damage and Elevated Production of Reactive Oxygen Species in PLHC‐1 Fish Cell Line,” Chemosphere 90 (2013): 1201–1209. 23121984 10.1016/j.chemosphere.2012.09.039 PMC4351966

[70] D. Ospondpant , S. Phuagkhaopong , K. Suknuntha , et al., “Cadmium Induces Apoptotic Program Imbalance and Cell Cycle Inhibitor Expression in Cultured Human Astrocytes,” Environmental Toxicology and Pharmacology 65 (2019): 53–59. 30537571 10.1016/j.etap.2018.12.001

[71] I. Pellarin , A. Dall'Acqua , A. Favero , et al., “Cyclin‐Dependent Protein Kinases and Cell Cycle Regulation in Biology and Disease,” Signal Transduction and Targeted Therapy 10 (2025): 11. 39800748 10.1038/s41392-024-02080-z PMC11734941

[72] B. Kang , J. Wang , and S. Guo , L. Yang , “Mercury‐Induced Toxicity: Mechanisms, Molecular Pathways, and Gene Regulation,” The Science of the Total Environment 943 (2024): 173577. 38852866 10.1016/j.scitotenv.2024.173577

[73] C. G. Yedjou , “Tchounwou PB: DNA Damage, Cell Cycle Arrest, and Apoptosis Induction Caused by Lead in Human Leukemia Cells,” International Journal of Environmental Research and Public Health 13 (2015): ijerph13010056. 26703663 10.3390/ijerph13010056 PMC4730447

[74] M. Ahamed and M. K. Siddiqui , “Low Level Lead Exposure and Oxidative Stress: Current Opinions,” Clinica Chimica Acta 383 (2007): 57–64. 10.1016/j.cca.2007.04.024 17573057

[75] A. Chamoli and S. K. Karn , “,The Effects of Mercury Exposure on Neurological and Cognitive Dysfunction in Human: A Review,” in Mercury Toxicity Mitigation: Sustainable Nexus Approach. ed. N. Kumar (Springer Nature Switzerland, 2024): 117–135.

[76] P. Deng , H. Zhang , L. Wang , et al., “Long‐Term Cadmium Exposure Impairs Cognitive Function by Activating lnc‐Gm10532/m6A/FIS1 Axis‐Mediated Mitochondrial Fission and Dysfunction,” The Science of the Total Environment 858 (2023): 159950. 36336035 10.1016/j.scitotenv.2022.159950

[77] S. R. Fan , T. T. Ren , M. Y. Yun , R. Lan , and X. Y. Qin , “Edaravone Attenuates Cadmium‐Induced Toxicity by Inhibiting Oxidative Stress and Inflammation in ICR Mice,” Neurotoxicology 86 (2021): 1–9. 34174317 10.1016/j.neuro.2021.06.003

[78] F. Xu , S. Farkas , S. Kortbeek , F. X. Zhang , L. Chen , and G. W. Zamponi , “Syed NI: Mercury‐Induced Toxicity of rat Cortical Neurons is Mediated Through N‐Methyl‐D‐Aspartate Receptors,” Molecular Brain 5 (2012): 30. 22980357 10.1186/1756-6606-5-30 PMC3462706

[79] A. H. Rezvani , “Involvement of the NMDA System in Learning and Memory,” In: Edited by ED Levin , JJ Buccafusco (CRC Press/Taylor & Francis, 2006). 21204373

[80] V. Karri , M. Schuhmacher , and V. Kumar , “Heavy Metals (Pb, Cd, As and MeHg) as Risk Factors for Cognitive Dysfunction: A General Review of Metal Mixture Mechanism in Brain,” Environmental Toxicology and Pharmacology 48 (2016): 203–213. 27816841 10.1016/j.etap.2016.09.016

[81] G. G. Kovacs , “Concepts and Classification of Neurodegenerative Diseases,” Handbook of Clinical Neurology 145 (2017): 301–307. 28987178 10.1016/B978-0-12-802395-2.00021-3

[82] I. Y. Iskusnykh , A. A. Zakharova , E. D. Kryl'skii , and T. N. Popova , “Aging, Neurodegenerative Disorders, and Cerebellum,” International Journal of Molecular Sciences 25 (2024): 1018. 38256091 10.3390/ijms25021018 PMC10815822

[83] G. S. Bloom , “Amyloid‐β and tau: The Trigger and Bullet in Alzheimer Disease Pathogenesis,” JAMA Neurology 71 (2014): 505–508. 24493463 10.1001/jamaneurol.2013.5847 PMC12908160

[84] L. Stefanis , “α‐Synuclein in Parkinson's Disease,” Cold Spring Harbor Perspectives in Medicine 2 (2012): a009399. 22355802 10.1101/cshperspect.a009399 PMC3281589

[85] E. Feneberg , E. Gray , O. Ansorge , K. Talbot , and M. R. Turner , “Towards a TDP‐43‐Based Biomarker for ALS and FTLD,” Molecular Neurobiology 55 (2018): 7789–7801. 29460270 10.1007/s12035-018-0947-6 PMC6132775

[86] C. A. Ross and S. J. Tabrizi , “Huntington's Disease: From Molecular Pathogenesis to Clinical Treatment,” The Lancet Neurology 10 (2011): 83–98. 21163446 10.1016/S1474-4422(10)70245-3

[87] K. Panchal and A. K. Tiwari , “Mitochondrial Dynamics, a Key Executioner in Neurodegenerative Diseases,” Mitochondrion 47 (2019): 151–173. 30408594 10.1016/j.mito.2018.11.002

[88] S. A. Wolf , H. W. Boddeke , and H. Kettenmann , “Microglia in Physiology and Disease,” Annual Review of Physiology 79 (2017): 619–643. 10.1146/annurev-physiol-022516-034406 27959620

[89] M. Lv , X. Ma , K. Zhang , et al., “The Disruption of Blood‐Brain Barrier Induced by Long‐Term Arsenic Exposure is Associated With the Increase of MMP‐9 and MMP‐2: The Characteristics are Similar to Those Caused by Senescence,” Chemico‐Biological Interactions 385 (2023): 110743. 37802410 10.1016/j.cbi.2023.110743

[90] T. Zhou , Z. Huang , X. Sun , et al., “Microglia Polarization With M1/M2 Phenotype Changes in rd1 Mouse Model of Retinal Degeneration,” Frontiers in Neuroanatomy 11 (2017): 77. 28928639 10.3389/fnana.2017.00077 PMC5591873

[91] J. A. Ansari , S. K. Mishra , R. K. Dey , et al., “Minocycline Reverses Developmental Arsenic Exposure‐Induced Microglia Activation and Functional Alteration in BALB/c Mice,” Environmental Toxicology and Pharmacology 92 (2022): 103858. 35351628 10.1016/j.etap.2022.103858

[92] R. Sharma , M. D. Abubakar , P. Bisht , et al., “Arsenic Exposure and Amyloid Precursor Protein Processing: A Focus on Alzheimer's Disease,” Current Molecular Pharmacology (2023) 10.2174/0118761429272806231020045840 37921143

[93] M. A. Rahman , M. A. Hannan , M. J. Uddin , M. S. Rahman , M. M. Rashid , and B. Kim , “Exposure to Environmental Arsenic and Emerging Risk of Alzheimer's Disease: Perspective Mechanisms, Management Strategy, and Future Directions,” Toxics 9 (2021): 188. 34437506 10.3390/toxics9080188 PMC8402411

[94] A. Chakraborty , S. Ghosh , B. Biswas , S. Pramanik , J. Nriagu , and S. Bhowmick , “Epigenetic Modifications From Arsenic Exposure: A Comprehensive Review,” The Science of the Total Environment 810 (2022): 151218. 34717984 10.1016/j.scitotenv.2021.151218

[95] J. Y. Min and K. B. Min , “Blood Cadmium Levels and Alzheimer's Disease Mortality Risk in Older US Adults,” Environmental Health : a Global Access Science Source 15 (2016): 69. 27301955 10.1186/s12940-016-0155-7 PMC4908725

[96] T. Ali , A. Khan , S. I. Alam , et al., “Cadmium, an Environmental Contaminant, Exacerbates Alzheimer's Pathology in the Aged Mice's Brain,” Frontiers in Aging Neuroscience 13 (2021): 650930. 34248598 10.3389/fnagi.2021.650930 PMC8263901

[97] J. Del Pino , G. Zeballos , M. J. Anadón , et al., “Cadmium‐Induced Cell Death of Basal Forebrain Cholinergic Neurons Mediated by Muscarinic M1 Receptor Blockade, Increase in GSK‐3β Enzyme, β‐Amyloid and Tau Protein Levels,” Archives of Toxicology 90 (2016): 1081–1092. 26026611 10.1007/s00204-015-1540-7

[98] T. Kimura , K. Ishiguro , and S. Hisanaga , “Physiological and Pathological Phosphorylation of Tau by CDK5,” Frontiers in Molecular Neuroscience 7 (2014): 65. 25076872 10.3389/fnmol.2014.00065 PMC4097945

[99] S. Phuagkhaopong , D. Ospondpant , T. Kasemsuk , et al., “Cadmium‐Induced IL‐6 and IL‐8 Expression and Release From Astrocytes are Mediated by MAPK and NF‐κB Pathways,” Neurotoxicology 60 (2017): 82–91. 28288823 10.1016/j.neuro.2017.03.001

[100] R. Siblerud , J. Mutter , E. Moore , J. Naumann , and H. Walach , “A Hypothesis and Evidence That Mercury May be an Etiological Factor in Alzheimer's Disease,” International Journal of Environmental Research and Public Health 16 (2019): 5152. 31861093 10.3390/ijerph16245152 PMC6950077

[101] L. Behzadfar , S. Hassani , H. Feizpour , et al., “Effects of Mercuric Chloride on Spatial Memory Deficit‐Induced by Beta‐Amyloid and Evaluation of Mitochondrial Function Markers in the Hippocampus of Rats,” Metallomics : Integrated Biometal Science 12 (2020): 144–153. 31793599 10.1039/c9mt00161a

[102] C. C. Zhou , Z. Y. Gao , J. Wang , et al., “Lead Exposure Induces Alzheimers's Disease (AD)‐Like Pathology and Disturbes Cholesterol Metabolism in the Young Rat Brain,” Toxicology Letters 296 (2018): 173–183. 29908845 10.1016/j.toxlet.2018.06.1065

[103] J. Wu , M. R. Basha , B. Brock , et al., “Alzheimer's Disease (AD)‐Like Pathology in Aged Monkeys After Infantile Exposure to Environmental Metal Lead (Pb): Evidence for a Developmental Origin and Environmental Link for AD,” The Journal of Neuroscience 28 (2008): 3–9. 18171917 10.1523/JNEUROSCI.4405-07.2008 PMC2486412

[104] A. Ashok , N. K. Rai , S. Tripathi , and S. Bandyopadhyay , “Exposure to As‐, Cd‐, and Pb‐Mixture Induces Aβ, Amyloidogenic APP Processing and Cognitive Impairments via Oxidative Stress‐Dependent Neuroinflammation in Young Rats,” Toxicological Sciences 143 (2015): 64–80. 25288670 10.1093/toxsci/kfu208

[105] P. Srivastava , Y. K. Dhuriya , R. Gupta , et al., “Protective Effect of Curcumin by Modulating BDNF/DARPP32/CREB in Arsenic‐Induced Alterations in Dopaminergic Signaling in Rat Corpus Striatum,” Molecular Neurobiology 55 (2018): 445–461. 27966075 10.1007/s12035-016-0288-2

[106] M. Golpich , E. Amini , F. Hemmati , et al., “Glycogen Synthase Kinase‐3 Beta (GSK‐3β) Signaling: Implications for Parkinson's Disease,” Pharmacological Research 97 (2015): 16–26. 25829335 10.1016/j.phrs.2015.03.010

[107] Y. Xu , H. Hong , X. Lin , et al., “Chronic Cadmium Exposure Induces Parkinson‐Like Syndrome by Eliciting Sphingolipid Disturbance And Neuroinflammation in the Midbrain of C57BL/6J Mice,” Environmental Pollution 337 (2023): 122606. 37742865 10.1016/j.envpol.2023.122606

[108] A. N. Tsentsevitsky and A. M. Petrov , “Synaptic Mechanisms of Cadmium Neurotoxicity,” Neural Regeneration Research 16 (2021): 1762–1763. 33510066 10.4103/1673-5374.306067 PMC8328762

[109] S. B. Mimouna , M. Chemek , S. Boughammoura , M. Banni , and I. Messaoudi , “Early‐Life Exposure to Cadmium Triggers Distinct Zn‐Dependent Protein Expression Patterns and Impairs Brain Development,” Biological Trace Element Research 184 (2018): 409–421. 29164515 10.1007/s12011-017-1201-1

[110] R. Pamphlett and D. P. Bishop , “Mercury is Present in Neurons and Oligodendrocytes in Regions of the Brain Affected by Parkinson's Disease and Co‐Localises With Lewy Bodies,” PLoS ONE 17 (2022): e0262464. 35015796 10.1371/journal.pone.0262464 PMC8752015

[111] V. Matović , A. Buha , D. Ðukić‐Ćosić , and Z. Bulat , “Insight Into the Oxidative Stress Induced by Lead and/or Cadmium in Blood, Liver and Kidneys,” Food and Chemical Toxicology 78 (2015): 130–140. 25681546 10.1016/j.fct.2015.02.011

[112] J. T. Rogers , V. Venkataramani , C. Washburn , et al., “A Role for Amyloid Precursor Protein Translation to Restore Iron Homeostasis and Ameliorate Lead (Pb) Neurotoxicity,” Journal of Neurochemistry 138 (2016): 479–494. 27206843 10.1111/jnc.13671

[113] J. Zhang , T. Cai , F. Zhao , et al., “The Role of α‐Synuclein and Tau Hyperphosphorylation‐Mediated Autophagy and Apoptosis in Lead‐Induced Learning and Memory Injury,” International Journal of Biological Sciences 8 (2012): 935–944. 22811615 10.7150/ijbs.4499 PMC3399316

[114] V. Gupta , N. G. Ansari , R. K. Garg , and S. Khattri , “Determination of Cd, Cr, Pb and Ni Contents Among Parkinson's Disease Individuals: A Case‐Control Study,” The International Journal of Neuroscience 127 (2017): 770–775. 27819176 10.1080/00207454.2016.1251917

[115] K. Rezaei , G. Mastali , E. Abbasgholinejad , et al., “Cadmium Neurotoxicity: Insights Into Behavioral Effect and Neurodegenerative Diseases,” Chemosphere 364 (2024): 143180. 39187026 10.1016/j.chemosphere.2024.143180

[116] M. Forcella , P. Lau , M. Oldani , et al., “Neuronal Specific and Non‐Specific Responses to Cadmium Possibly Involved in Neurodegeneration: A Toxicogenomics Study in a Human Neuronal Cell Model,” Neurotoxicology 76 (2020): 162–173. 31738976 10.1016/j.neuro.2019.11.002

[117] B. Xu , S. Chen , Y. Luo , et al., “Calcium Signaling is Involved in Cadmium‐Induced Neuronal Apoptosis via Induction of Reactive Oxygen Species and Activation of MAPK/mTOR Network,” PLoS ONE 6 (2011): e19052. 21544200 10.1371/journal.pone.0019052 PMC3081326

[118] Y. Yuan , C. Y. Jiang , H. Xu , et al., “Cadmium‐Induced Apoptosis in Primary rat Cerebral Cortical Neurons Culture is Mediated by a Calcium Signaling Pathway,” PLoS ONE 8 (2013): e64330. 23741317 10.1371/journal.pone.0064330 PMC3669330

[119] Y. H. Huang , C. M. Shih , C. J. Huang , et al., “Effects of Cadmium on Structure and Enzymatic Activity of Cu,Zn‐SOD and Oxidative Status in Neural Cells,” Journal of Cellular Biochemistry 98 (2006): 577–589. 16440303 10.1002/jcb.20772

[120] A. Prasad , V. Bharathi , V. Sivalingam , A. Girdhar , and B. K. Patel , “Molecular Mechanisms of TDP‐43 Misfolding and Pathology in Amyotrophic Lateral Sclerosis,” Frontiers in Molecular Neuroscience 12 (2019): 25. 30837838 10.3389/fnmol.2019.00025 PMC6382748

[121] E. Minj , S. Upadhayay , and S. Mehan , “Nrf2/HO‐1 Signaling Activator Acetyl‐11‐Keto‐Beta Boswellic Acid (AKBA)‐Mediated Neuroprotection in Methyl Mercury‐Induced Experimental Model of ALS,” Neurochemical Research 46 (2021): 2867–2884. 34075522 10.1007/s11064-021-03366-2

[122] P. E. A. Ash , U. Dhawan , S. Boudeau , et al., “Heavy Metal Neurotoxicants Induce ALS‐Linked TDP‐43 Pathology,” Toxicological Sciences 167 (2019): 105–115. 30371865 10.1093/toxsci/kfy267 PMC6317426

[123] A. Shandilya , S. Mehan , S. Kumar , et al., “Activation of IGF‐1/GLP‐1 Signalling via 4‐Hydroxyisoleucine Prevents Motor Neuron Impairments in Experimental ALS‐Rats Exposed to Methylmercury‐Induced Neurotoxicity,” Molecules (Basel, Switzerland)27 (2022): 3878. 35745001 10.3390/molecules27123878 PMC9228431

[124] M. R. Vargas , M. Pehar , P. J. Díaz‐Amarilla , J. S. Beckman , and L. Barbeito , “Transcriptional Profile of Primary Astrocytes Expressing ALS‐Linked Mutant SOD1,” Journal of Neuroscience Research 86 (2008): 3515–3525. 18683239 10.1002/jnr.21797 PMC4048747

[125] L. C. Wijesekera and P. N. Leigh , “Amyotrophic Lateral Sclerosis,” Orphanet Journal of Rare Diseases 4 (2009): 3. 19192301 10.1186/1750-1172-4-3 PMC2656493

[126] A. Chiò , A. Calvo , L. Mazzini , et al., “Extensive Genetics of ALS: A Population‐Based Study in Italy,” Neurology 79 (2012): 1983–1989. 23100398 10.1212/WNL.0b013e3182735d36 PMC3484987

[127] M. S. Rotunno , D. A. Bosco , “An Emerging Role for Misfolded Wild‐Type SOD1 in Sporadic ALS Pathogenesis,” Frontiers in Cellular Neuroscience 7 (2013): 253. 24379756 10.3389/fncel.2013.00253 PMC3863749

[128] D. R. Rosen , T. Siddique , D. Patterson , et al., “Mutations in Cu/Zn Superoxide Dismutase Gene are Associated With Familial Amyotrophic Lateral Sclerosis,” Nature 362 (1993): 59–62. 8446170 10.1038/362059a0

[129] P. J. Kamitsuka , M. M. Ghanem , R. Ziar , S. E. McDonald , M. G. Thomas , and G. F. Kwakye , “Defective Mitochondrial Dynamics and Protein Degradation Pathways Underlie Cadmium‐Induced Neurotoxicity and Cell Death in Huntington's Disease Striatal Cells,” International Journal of Molecular Sciences 24 (2023): 7178. 37108341 10.3390/ijms24087178 PMC10139096

[130] G. F. Kwakye , J. A. Jiménez , M. G. Thomas , et al., “Heterozygous Huntingtin Promotes Cadmium Neurotoxicity and Neurodegeneration in Striatal Cells via Altered Metal Transport and Protein Kinase C Delta Dependent Oxidative Stress and Apoptosis Signaling Mechanisms,” Neurotoxicology 70 (2019): 48–61. 30399392 10.1016/j.neuro.2018.10.012

[131] Y. H. Wu , J. C. Lin , T. Y. Wang , et al., “Hexavalent Chromium Intoxication Induces Intrinsic and Extrinsic Apoptosis in Human Renal Cells,” Molecular Medicine Reports 21 (2020): 851–857. 31974625 10.3892/mmr.2019.10885 PMC6947900

[132] B. A. Fowler , “14‐Arsenical Kidney Toxicity,” Handbook of Arsenic Toxicology (2023): 395–408.

[133] S. Satarug , “Is Chronic Kidney Disease Due to Cadmium Exposure Inevitable and Can It Be Reversed?” Biomedicines 12 (2024): 718. 38672074 10.3390/biomedicines12040718 PMC11048639

[134] Y. Rbaibi , K. R. Long , K. E. Shipman , et al., “Megalin, Cubilin, and Dab2 Drive Endocytic Flux in Kidney Proximal Tubule Cells,” Molecular Biology of the Cell 34 (2023): ar74. 37126375 10.1091/mbc.E22-11-0510 PMC10295476

[135] X. Wang , L. Han , G. Li , et al., “From the Cover: Identification of Natural Products as Inhibitors of Human Organic Anion Transporters (OAT1 and OAT3) and Their Protective Effect on Mercury‐Induced Toxicity,” Toxicological Sciences 161 (2018): 321–334. 29045746 10.1093/toxsci/kfx216

[136] C. C. Bridges and R. K. Zalups , “The Aging Kidney and the Nephrotoxic Effects of Mercury,” Journal of Toxicology and Environmental Health Part B, Critical Reviews 20 (2017): 55–80. 28339347 10.1080/10937404.2016.1243501 PMC6088787

[137] R. K. Zalups , “Molecular Interactions With Mercury in the Kidney,” Pharmacological Reviews 52 (2000): 113–143. 10699157

[138] M. N. Rana , J. Tangpong , and M. M. Rahman , “Toxicodynamics of Lead, Cadmium, Mercury and Arsenic‐ Induced Kidney Toxicity and Treatment Strategy: A Mini Review,” Toxicology Reports 5 (2018): 704–713. 29992094 10.1016/j.toxrep.2018.05.012 PMC6035907

[139] C. V. Nolan and Z. A. Shaikh , “Lead Nephrotoxicity and Associated Disorders: Biochemical Mechanisms,” Toxicology 73 (1992): 127–146. 1319092 10.1016/0300-483x(92)90097-x

[140] Z. Pan , T. Gong , and P. Liang , “Heavy Metal Exposure and Cardiovascular Disease,” Circulation Research 134 (2024): 1160–1178. 38662861 10.1161/CIRCRESAHA.123.323617

[141] M. C. Sanguinetti , C. Jiang , M. E. Curran , and M. T. Keating , “A Mechanistic Link Between an Inherited and an Acquired Cardiac Arrhythmia: HERG Encodes the IKr Potassium Channel,” Cell 81 (1995): 299–307. 7736582 10.1016/0092-8674(95)90340-2

[142] J. L. Mumford , K. Wu , Y. Xia , et al., “Chronic Arsenic Exposure and Cardiac Repolarization Abnormalities With QT Interval Prolongation in a Population‐Based Study,” Environmental Health Perspectives 115 (2007): 690–694. 17520054 10.1289/ehp.9686 PMC1867981

[143] X. Cao , S. Wang , R. Bi , S. Tian , Y. Huo , and J. Liu , “Toxic Effects of Cr(VI) on the Bovine Hemoglobin and Human Vascular Endothelial Cells: Molecular Interaction and Cell Damage,” Chemosphere 222 (2019): 355–363. 30710761 10.1016/j.chemosphere.2019.01.137

[144] G. Choong , Y. Liu , and D. M. Templeton , “Interplay of Calcium and Cadmium in Mediating Cadmium Toxicity,” Chemico‐Biological Interactions 211 (2014): 54–65. 24463198 10.1016/j.cbi.2014.01.007

[145] M. T. Nelson , “Interactions of Divalent Cations With Single Calcium Channels From Rat Brain Synaptosomes,” Journal of General Physiology 87 (1986): 201–222. 2419482 10.1085/jgp.87.2.201 PMC2217603

[146] N. B. Lemos , J. K. Angeli , O. Faria Tde , et al., “Low Mercury Concentration Produces Vasoconstriction, Decreases Nitric Oxide Bioavailability and Increases Oxidative Stress in Rat Conductance Artery,” PLoS ONE 7 (2012): e49005. 23145049 10.1371/journal.pone.0049005 PMC3492199

[147] Z. W. Hu , R. Kerb , X. Y. Shi , T. Wei‐Lavery , and B. B. Hoffman , “Angiotensin II Increases Expression of Cyclooxygenase‐2: Implications for the Function of Vascular Smooth Muscle Cells,” The Journal of Pharmacology and Experimental Therapeutics 303 (2002): 563–573. 12388637 10.1124/jpet.102.037705

[148] T. O. Faria , M. R. Simões , D. V. Vassallo , et al., “Xanthine Oxidase Activation Modulates the Endothelial (Vascular) Dysfunction Related to HgCl(2) Exposure Plus Myocardial Infarction in Rats,” Cardiovascular Toxicology 18 (2018): 161–174. 28980197 10.1007/s12012-017-9427-x

[149] N. D. Vaziri , “Mechanisms of Lead‐Induced Hypertension and Cardiovascular Disease,” American Journal of Physiology. Heart and Circulatory Physiology 295 (2008): H454–465. 18567711 10.1152/ajpheart.00158.2008 PMC2519216

[150] J. J. Wellings , J. M. Thorpe , K. Yendole , Y. Matsubayashi , and P. S. Hartley , “Effect of Short and Long‐Term Cadmium Exposure on Behaviour and Cardiac Function in Drosophila,” Environmental Pollution (Barking, Essex : 1987) 366 (2025): 125481. 39644948 10.1016/j.envpol.2024.125481

[151] M. L. Fitch , R. Kabir , O. V. Ebenebe , et al., “Cadmium Exposure Induces a Sex‐Dependent Decline in Left Ventricular Cardiac Function,” Life Sciences 324 (2023): 121712. 37100378 10.1016/j.lfs.2023.121712 PMC10246466

[152] Q. Liu , C. Xu , J. Jin , et al., “Early‐Life Exposure to Lead Changes Cardiac Development and Compromises Long‐Term Cardiac Function,” The Science of the Total Environment 904 (2023): 166667. 37652374 10.1016/j.scitotenv.2023.166667

[153] G. Ferreira de Mattos , C. Costa , F. Savio , M. Alonso , and G. L. Nicolson , “Lead Poisoning: Acute Exposure of the Heart to Lead Ions Promotes Changes in Cardiac Function and Cav1.2 ion Channels,” Biophysical Reviews 9 (2017): 807–825. 28836190 10.1007/s12551-017-0303-5 PMC5662044

[154] R. Balakrishnan , C. S. Kumar , M. U. Rani , K. Kavita , G. Boobalan , and A. G. Reddy , “Evaluation of Protective Action of α‐Tocopherol in Chromium‐Induced Oxidative Stress in Female Reproductive System of Rats,” Journal of Natural Science, Biology and Medicine 4 (2013): 87–93. 23633841 10.4103/0976-9668.107266 PMC3633310

[155] A. K. Chandra , A. Chatterjee , R. Ghosh , and M. Sarkar , “Effect of Curcumin on Chromium‐Induced Oxidative Damage in Male Reproductive System,” Environmental Toxicology and Pharmacology 24 (2007): 160–166. 21783805 10.1016/j.etap.2007.04.009

[156] P. Chen , Q. Luo , Y. Lin , et al., “Arsenic Exposure During Juvenile and Puberty Significantly Affected Reproductive System Development of Female SD Rats,” Ecotoxicology and Environmental Safety 242 (2022): 113857. 35809398 10.1016/j.ecoenv.2022.113857

[157] S. Mitra , S. Bhattacharyya , S. Ray , et al., “Resveratrol Alleviates Cadmium‐Induced Damage and Overexpression of Epidermal Growth Factor Receptor and its Downstream Signaling Proteins in the Reproductive System of Male Swiss Albino Mice,” Journal of Environmental Pathology, Toxicology and Oncology 35 (2016): 73–90. 10.1615/JEnvironPatholToxicolOncol.2016015298 27279585

[158] S. Li , B. Han , P. Wu , et al., “Effect of Inorganic Mercury Exposure on Reproductive System of male Mice: Immunosuppression and Fibrosis in Testis,” Environmental Toxicology 37 (2022): 69–78. 34569128 10.1002/tox.23378

[159] M. M. Ommati , H. N. Ahmadi , S. Sabouri , et al., “Glycine Protects the Male Reproductive System Against Lead Toxicity via Alleviating Oxidative Stress, Preventing Sperm Mitochondrial Impairment, Improving Kinematics of Sperm, and Blunting the Downregulation of Enzymes Involved in the Steroidogenesis,” Environmental Toxicology 37 (2022): 2990–3006. 36088639 10.1002/tox.23654

[160] P. Jacquet and J. P. Draye , “Toxicity of Chromium Salts to Cultured Mouse Embryos,” Toxicology Letters 12 (1982): 53–57. 7112602 10.1016/0378-4274(82)90198-9

[161] C. Zhang , C. Liu , D. Li , et al., “Intracellular Redox Imbalance and Extracellular Amino Acid Metabolic Abnormality Contribute to Arsenic‐Induced Developmental Retardation in Mouse Preimplantation Embryos,” Journal of Cellular Physiology 222 (2010): 444–455. 19918794 10.1002/jcp.21966

[162] J. Zhu , Z. Huang , F. Yang , et al., “Cadmium Disturbs Epigenetic Modification and Induces DNA Damage in Mouse Preimplantation Embryos,” Ecotoxicology and Environmental Safety 219 (2021): 112306. 33984557 10.1016/j.ecoenv.2021.112306

[163] W. Yu , Y. Tu , Z. Long , et al., “Reactive Oxygen Species Bridge the Gap Between Chronic Inflammation and Tumor Development,” Oxidative Medicine and Cellular Longevity 2022 (2022): 2606928. 35799889 10.1155/2022/2606928 PMC9256443

[164] L. Zhao , R. Islam , Y. Wang , X. Zhang , and L. Z. Liu , “Epigenetic Regulation in Chromium‐, Nickel‐ and Cadmium‐Induced Carcinogenesis,” Cancers 14 (2022): 5768. 36497250 10.3390/cancers14235768 PMC9737485

[165] Z. Feng , W. Hu , W. N. Rom , M. Costa , and M. S. Tang , “Chromium(VI) Exposure Enhances Polycyclic Aromatic Hydrocarbon‐DNA Binding at the p53 Gene in Human Lung Cells,” Carcinogenesis 24 (2003): 771–778. 12727806 10.1093/carcin/bgg012

[166] C. Ren , Y. Zhou , W. Liu , and Q. Wang , “Paradoxical Effects of Arsenic in the Lungs,” Environmental Health and Preventive Medicine 26 (2021): 80. 34388980 10.1186/s12199-021-00998-2 PMC8364060

[167] R. Hubaux , D. D. Becker‐Santos , K. S. Enfield , S. Lam , W. L. Lam , and V. D. Martinez , “Arsenic, Asbestos and Radon: Emerging Players in Lung Tumorigenesis,” Environmental Health 11 (2012): 89. 23173984 10.1186/1476-069X-11-89 PMC3534001

[168] R. J. Person , E. J. Tokar , Y. Xu , R. Orihuela , N. N. Ngalame , and M. P. Waalkes , “Chronic Cadmium Exposure in Vitro Induces Cancer Cell Characteristics in Human Lung Cells,” Toxicology and Applied Pharmacology 273 (2013): 281–288. 23811327 10.1016/j.taap.2013.06.013 PMC3863781

[169] C. L. Siewit , B. Gengler , E. Vegas , R. Puckett , and M. C. Louie , “Cadmium Promotes Breast Cancer Cell Proliferation by Potentiating the Interaction Between ERalpha and c‐Jun,” Molecular Endocrinology (Baltimore, Md) 24 (2010): 981–992. 20219890 10.1210/me.2009-0410 PMC2870938

[170] X. Yu , E. J. Filardo , and Z. A. Shaikh , “The Membrane Estrogen Receptor GPR30 Mediates Cadmium‐Induced Proliferation of Breast Cancer Cells,” Toxicology and Applied Pharmacology 245 (2010): 83–90. 20153348 10.1016/j.taap.2010.02.005

[171] G. Furtak , M. Kozłowski , S. Kwiatkowski , and A. Cymbaluk‐Płoska , “The Role of Lead and Cadmium in Gynecological Malignancies,” Antioxidants (Basel, Switzerland) 11 (2022): 2468. 36552675 10.3390/antiox11122468 PMC9774668

[172] A. Stoica , B. S. Katzenellenbogen , and M. B. Martin , “Activation of Estrogen Receptor‐Alpha by the Heavy Metal Cadmium,” Molecular Endocrinology (Baltimore, Md) 14 (2000): 545–553. 10770491 10.1210/mend.14.4.0441

[173] C. P. Pineda‐Belmontes , R. U. Hernández‐Ramírez , C. Hernández‐Alcaraz , M. E. Cebrián , and L. López‐Carrillo , “Genetic Polymorphisms of PPAR Gamma, Arsenic Methylation Capacity and Breast Cancer Risk in Mexican Women,” Salud Publica De Mexico 58 (2016): 220–227. 27557380 10.21149/spm.v58i2.7791

[174] J. D. Davis and S. Y. Lin , “DNA Damage and Breast Cancer,” World Journal of Clinical Oncology 2 (2011): 329–338. 21909479 10.5306/wjco.v2.i9.329 PMC3168783

[175] A. Nersesyan , M. Kundi , M. Waldherr , et al., “Results of Micronucleus Assays With Individuals Who Are Occupationally and Environmentally Exposed to Mercury, Lead and Cadmium,” Mutation Research Reviews in Mutation Research 770 (2016): 119–139. 27894681 10.1016/j.mrrev.2016.04.002

[176] W. Qu , H. Ke , J. Pi , et al., “Acquisition of Apoptotic Resistance in Cadmium‐Transformed Human Prostate Epithelial Cells: BCL‐2 Overexpression Blocks the Activation of JNK Signal Transduction Pathway,” Environmental Health Perspectives 115 (2007): 1094–1100. 17637928 10.1289/ehp.10075 PMC1913575

[177] L. Benbrahim‐Tallaa and M. P. Waalkes , “Inorganic Arsenic and Human Prostate Cancer,” Environmental Health Perspectives 116 (2008): 158–164. 18288312 10.1289/ehp.10423 PMC2235216

[178] L. Benbrahim‐Tallaa , R. A. Waterland , M. Styblo , W. E. Achanzar , M. M. Webber , and M. P. Waalkes , “Molecular Events Associated With Arsenic‐Induced Malignant Transformation of Human Prostatic Epithelial Cells: Aberrant Genomic DNA Methylation and K‐Ras Oncogene Activation,” Toxicology and Applied Pharmacology 206 (2005): 288–298. 16039940 10.1016/j.taap.2004.11.017

[179] M. Sun , Z. Jiang , P. Gu , et al., “Cadmium Promotes Colorectal Cancer Metastasis Through EGFR/Akt/mTOR Signaling Cascade and Dynamics,” The Science of the Total Environment 899 (2023): 165699. 37495125 10.1016/j.scitotenv.2023.165699

[180] Z. Zhang , H. Cao , N. Song , L. Zhang , Y. Cao , and J. Tai , “Long‐Term Hexavalent Chromium Exposure Facilitates Colorectal Cancer in Mice Associated With Changes in Gut Microbiota Composition,” Food and Chemical Toxicology 138 (2020): 111237. 32145354 10.1016/j.fct.2020.111237

[181] M. Zemelka‐Wiacek , “The Interaction Among Effector, Regulatory, and Tγδ Cells Determines the Development of Allergy or Tolerance to Chromium,” Journal of Clinical Medicine 14 (2025): 1370. 40004900 10.3390/jcm14041370 PMC11856200

[182] Q. Qian , P. Li , T. Wang , et al., “Alteration of Th1/Th2/Th17 Cytokine Profile and Humoral Immune Responses Associated With Chromate Exposure,” Occupational and Environmental Medicine 70 (2013): 697–702. 23811143 10.1136/oemed-2013-101421

[183] L. N. Islam : 21‐An Update on the Immunotoxic Effects of Arsenic Exposure. Handbook of Arsenic Toxicology 2nd Edn. In: Edited by Flora SJS. (Oxford: Academic Press, 2023): 551–592.

[184] I. Mirkov , A. Popov Aleksandrov , and M. Ninkov , “Immunotoxicology of Cadmium: Cells of the Immune System as Targets and Effectors of Cadmium Toxicity,” Food and Chemical Toxicology 149 (2021): 112026. 33508420 10.1016/j.fct.2021.112026

[185] N. Pathak and S. Khandelwal , “Impact of Cadmium in T Lymphocyte Subsets and Cytokine Expression: Differential Regulation by Oxidative Stress and Apoptosis,” Biometals 21 (2008): 179–187. 17641822 10.1007/s10534-007-9106-7

[186] K. M. Pollard , D. M. Cauvi , C. B. Toomey , P. Hultman , and D. H. Kono , “Mercury‐Induced Inflammation and Autoimmunity,” Biochimica Et Biophysica Acta General Subjects 1863 (2019): 129299. 30742953 10.1016/j.bbagen.2019.02.001 PMC6689266

[187] U. Undeger , N. Başaran , H. Canpinar , and E. Kansu , “Immune Alterations in Lead‐Exposed Workers,” Toxicology 109 (1996): 167–172. 8658547 10.1016/0300-483x(96)03333-1

[188] M. E. Sears , “Chelation: Harnessing and Enhancing Heavy Metal Detoxification–A Review,” The Scientific World Journal 2013 (2013): 219840. 23690738 10.1155/2013/219840 PMC3654245

[189] İ. Gulcin and S. H. Alwasel , “Metal Ions, Metal Chelators and Metal Chelating Assay as Antioxidant Method,” Processes 10 (2022): 132.

[190] W. Wu , L. Chen , W. Zhang , and D. Mei , “Comparison of Heavy Metal Contaminants Removal Using EDTA and Cyanex 302 as Chelating Agents for Supercritical CO2‐Based Soil Remediation,” Chemical Engineering Research and Design 206 (2024): 130–138.

[191] T. George and M. F. Brady , “Ethylenediaminetetraacetic Acid (EDTA),” StatPearls. (StatPearls Publishing, 2023). 33351441

[192] G. Bjørklund , G. Crisponi , V. M. Nurchi , R. Cappai , A. Buha Djordjevic , and J. Aaseth , “A Review on Coordination Properties of Thiol‐Containing Chelating Agents Towards Mercury, Cadmium, and Lead,” Molecules (Basel, Switzerland) 24 (2019): 3247. 31489907 10.3390/molecules24183247 PMC6767255

[193] A. Kovács , D. S. Nemcsok , and T. Kocsis , “Bonding Interactions in EDTA Complexes,” Journal of Molecular Structure 950 (2010): 93–97.

[194] R. A. Goyer , M. G. Cherian , M. M. Jones , and J. R. Reigart , “Role of Chelating Agents for Prevention, Intervention, and Treatment of Exposures to Toxic Metals,” Environmental Health Perspectives 103 (1995): 1048–1052. 8605855 10.1289/ehp.951031048 PMC1519187

[195] J. Aaseth , M. A. Skaug , Y. Cao , and O. Andersen , “Chelation in Metal Intoxication–Principles and Paradigms,” Journal of Trace Elements in Medicine and Biology 31 (2015): 260–266. 25457281 10.1016/j.jtemb.2014.10.001

[196] M. J. Kosnett , “The Role of Chelation in the Treatment of Arsenic and Mercury Poisoning,” Journal of Medical Toxicology 9 (2013): 347–354. 24178900 10.1007/s13181-013-0344-5 PMC3846971

[197] M. B. Sulzberger , R. L. Baer , and A. Kanof , “Clinical Uses of 2,3‐Dimercaptopropanol (BAL); Studies on the Toxicity of BAL on Percutaneous and Parenteral Administration,” The Journal of Clinical Investigation 25 (1946): 474–479. 21001622

[198] R. A. Peters , “Development and Theoretical Significance of British Anti‐Lewisite,” British Medical Bulletin 5 (1948): 313–319. 18892307 10.1093/oxfordjournals.bmb.a073250

[199] O. Andersen , “Principles and Recent Developments in Chelation Treatment of Metal Intoxication,” Chemical Reviews 99 (1999): 2683–2710. 11749497 10.1021/cr980453a

[200] M. J. Kosnett : BAL (DIMERCAPROL). Poisoning & Drug Overdose. 7e edn. In: Edited by Olson KR , Anderson IB , Benowitz NL , Blanc PD , Clark RF , Kearney TE , Kim‐Katz SY , Wu AHB (McGraw‐Hill Education, 2018).

[201] S. Bradberry and A. Vale , “Dimercaptosuccinic Acid (Succimer; DMSA) in Inorganic Lead Poisoning,” Clin Toxicol (Phila) 47 (2009): 617–631. 19663612 10.1080/15563650903174828

[202] A. L. Miller , “Dimercaptosuccinic Acid (DMSA), a Non‐Toxic, Water‐Soluble Treatment for Heavy Metal Toxicity,” Alternative Medicine Review 3 (1998): 199–207. 9630737

[203] H. V. Aposhian , R. M. Maiorino , R. C. Dart , and D. F. Perry , “Urinary Excretion of Meso‐2,3‐Dimercaptosuccinic Acid in Human Subjects,” Clinical Pharmacology & Therapeutics 45 (1989): 520–526. 2541962 10.1038/clpt.1989.67

[204] V. Gersl , R. Hrdina , J. Vávrová , et al., “Effects of Repeated Administration of Dithiol Chelating Agent–Sodium 2,3‐Dimercapto‐1‐Propanesulphonate (DMPS)–On Biochemical and Haematological Parameters in Rabbits,” Acta Medica (Hradec Kralove) 40 (1997): 3–8. 9329207

[205] S. J. Flora , R. Bhattacharya , and R. Vijayaraghavan , “Combined Therapeutic Potential of Meso‐2,3‐Dimercaptosuccinic Acid and Calcium Disodium Edetate on the Mobilization and Distribution of Lead in Experimental Lead Intoxication in Rats,” Fundamental and Applied Toxicology 25 (1995): 233–240. 7665007 10.1006/faat.1995.1059

[206] K. Kalia and S. J. Flora , “Strategies for Safe and Effective Therapeutic Measures for Chronic Arsenic and Lead Poisoning,” Journal of Occupational Health 47 (2005): 1–21. 10.1539/joh.47.1 15703449

[207] R. F. Kidd , “Results of Dental Amalgam Removal and Mercury Detoxification Using DMPS and Neural Therapy,” Alternative Therapies in Health and Medicine 6 (2000): 49–55. 10895513

[208] S. J. Flora and A. Mehta , “Monoisoamyl Dimercaptosuccinic Acid Abrogates Arsenic‐Induced Developmental Toxicity in Human Embryonic Stem Cell‐Derived Embryoid Bodies: Comparison With in Vivo Studies,” Biochemical Pharmacology 78 (2009): 1340–1349. 19615344 10.1016/j.bcp.2009.07.003

[209] M. M. Jones , P. K. Singh , G. R. Gale , A. B. Smith , and L. M. Atkins , “Cadmium Mobilization in Vivo by Intraperitoneal or Oral Administration of Monoalkyl Esters of Meso‐2,3‐Dimercaptosuccinic Acid in the Mouse,” Pharmacology & Toxicology 70 (1992): 336–343. 1319053 10.1111/j.1600-0773.1992.tb00483.x

[210] A. Mehta , S. C. Pant , and S. J. Flora , “Monoisoamyl Dimercaptosuccinic Acid Induced Changes in Pregnant Female Rats During Late Gestation and Lactation,” Reproductive Toxicology 21 (2006): 94–103. 16040228 10.1016/j.reprotox.2005.05.008

[211] M. W. Taubeneck , J. L. Domingo , J. M. Llobet , C. L. Keen , “Meso‐2,3‐Dimercaptosuccinic Acid (DMSA) Affects Maternal and Fetal Copper Metabolism in Swiss Mice,” Toxicology 72 (1992): 27–40. 1311466 10.1016/0300-483x(92)90083-q

[212] M. M. Jones , M. A. Basinger , G. R. Gale , L. M. Atkins , A. B. Smith , and A. Stone , “Effect of Chelate Treatments on Kidney, Bone and Brain Lead Levels of Lead‐Intoxicated Mice,” Toxicology 89 (1994): 91–100. 8197593 10.1016/0300-483x(94)90217-8

[213] S. K. Tandon , S. Singh , and V. K. Jain , “Efficacy of Combined Chelation in Lead Intoxication,” Chemical Research in Toxicology 7 (1994): 585–589. 7841335 10.1021/tx00041a001

[214] S. J. Flora , G. M. Kannan , B. P. Pant , and D. K. Jaiswal , “Combined Administration of Oxalic Acid, Succimer and its Analogue for the Reversal of Gallium Arsenide‐Induced Oxidative Stress in Rats,” Archives of Toxicology 76 (2002): 269–276. 12107644 10.1007/s00204-002-0347-5

[215] S. J. Flora , M. Pande , G. M. Kannan , and A. Mehta , “Lead Induced Oxidative Stress and its Recovery Following Co‐Administration of Melatonin or N‐Acetylcysteine During Chelation With Succimer in Male Rats,” Cellular and Molecular Biology 50 (2004): Ol543–551. 15555419

[216] A. Sharma , C. Kshetrimayum , H. G. Sadhu , and S. Kumar , “Arsenic‐Induced Oxidative Stress, Cholinesterase Activity in the Brain of Swiss Albino Mice, and its Amelioration by Antioxidants Vitamin E and Coenzyme Q10,” Environmental Science and Pollution Research International 25 (2018): 23946–23953. 29948670 10.1007/s11356-018-2398-z

[217] V. Eybl , J. Sýkora , J. Koutenský , D. Caisová , A. Schwartz , and F. Mertl , “Interaction of Chelating Agents With cadmium in Mice and Rats,” Environmental Health Perspectives 54 (1984): 267–273. 6734561 10.1289/ehp.8454267 PMC1568180

[218] M. Berlin and S. Ullrebg , “Increased Uptake of Mercury in Mouse Brain Caused by 2,3‐Dimercaptopropanol,” Nature 197 (1963): 84–85. 13970882 10.1038/197084a0

[219] S. M. Suru , “Onion and Garlic Extracts Lessen Cadmium‐Induced Nephrotoxicity in Rats,” Biometals : an International Journal on the Role of Metal Ions in Biology, Biochemistry, and Medicine 21 (2008): 623–633. 18521705 10.1007/s10534-008-9148-5

[220] S. K. Senapati , S. Dey , S. K. Dwivedi , and D. Swarup , “Effect of Garlic (Allium Sativum L.) Extract on Tissue Lead Level in Rats,” Journal of Ethnopharmacology 76 (2001): 229–232. 11448543 10.1016/s0378-8741(01)00237-9

[221] M. Aga , K. Iwaki , Y. Ueda , et al., “Preventive Effect of Coriandrum Sativum (Chinese Parsley) on Localized Lead Deposition in ICR Mice,” Journal of Ethnopharmacology 77 (2001): 203–208. 11535365 10.1016/s0378-8741(01)00299-9

[222] S. Petteruti , “Reduction of Lead Levels in Patients Following a Long‐Term, Intermittent Calcium Ethylenediaminetetraacetic Acid (EDTA)‐Based Intravenous Chelation Infusions: A Prospective Experimental Cohort,” Cureus 12 (2020): e11685. 33262921 10.7759/cureus.11685 PMC7689946

[223] S. Bradberry , T. Sheehan , and A. Vale , “Use of Oral Dimercaptosuccinic Acid (Succimer) in Adult Patients With Inorganic Lead Poisoning,” QJM : Monthly Journal of the Association of Physicians 102 (2009): 721–732. 19700440 10.1093/qjmed/hcp114

[224] G. Drasch , S. Böse‐O'Reilly , C. Beinhoff , G. Roider , and S. Maydl , “The Mt. Diwata Study on the Philippines 1999–Assessing Mercury Intoxication of the Population by Small Scale Gold Mining,” The Science of the Total Environment 267 (2001): 151–168. 11286210 10.1016/s0048-9697(00)00806-8

[225] S. S. Ali , H. Ahsan , M. K. Zia , T. Siddiqui , and F. H. Khan , “Understanding Oxidants and Antioxidants: Classical Team With New Players,” Journal of Food Biochemistry 44 (2020): e13145. 31960481 10.1111/jfbc.13145

[226] L. Patrick , “Toxic Metals and Antioxidants: Part II. The Role of Antioxidants in Arsenic and Cadmium Toxicity,” Alternative Medicine Review 8 (2003): 106–128. 12777158

[227] Z. Cao , M. Tsang , H. Zhao , and Y. Li , “Induction of Endogenous Antioxidants and Phase 2 Enzymes by α‐lipoic Acid in Rat Cardiac H9C2 Cells: Protection Against Oxidative Injury,” Biochemical and Biophysical Research Communications 310 (2003): 979–985. 14550301 10.1016/j.bbrc.2003.09.110

[228] E. Jiang , X. Chen , Y. Bi , C. Pan , X. Li , and X. Lan , “Curcumin Inhibits Oxidative Stress and Apoptosis Induced by H(2)O(2) in Bovine Adipose‐Derived Stem Cells (bADSCs),” Animals 14 (2024): 3421. 39682386 10.3390/ani14233421 PMC11640669

[229] A. T. Jan , M. Azam , K. Siddiqui , A. Ali , I. Choi , and Q. M. Haq , “Heavy Metals and Human Health: Mechanistic Insight Into Toxicity and Counter Defense System of Antioxidants,” International Journal of Molecular Sciences 16 (2015): 29592–29630. 26690422 10.3390/ijms161226183 PMC4691126

[230] J. J. Strain and C. W. Mulholland , “Vitamin C and Vitamin E–Synergistic Interactions in Vivo?” Experientia Supplementum 62 (1992): 419–422. 1450602 10.1007/978-3-0348-7460-1_40

[231] M. J. Ferreira , T. A. Rodrigues , A. G. Pedrosa , et al., “Glutathione and Peroxisome Redox Homeostasis,” Redox Biology 67 (2023): 102917. 37804696 10.1016/j.redox.2023.102917 PMC10565873

[232] G. Aldini , A. Altomare , G. Baron , et al., “N‐Acetylcysteine as an Antioxidant and Disulphide Breaking Agent: The Reasons Why,” Free Radical Research 52 (2018): 751–762. 29742938 10.1080/10715762.2018.1468564

[233] T. T. Joseph , V. Schuch , D. J. Hossack , R. Chakraborty , and E. L. Johnson , “Melatonin: The Placental Antioxidant and Anti‐Inflammatory,” Frontiers in Immunology 15 (2024): 1339304. 38361952 10.3389/fimmu.2024.1339304 PMC10867115

[234] I. Pérez‐Torres , V. Castrejón‐Téllez , M. E. Soto , M. E. Rubio‐Ruiz , L. Manzano‐Pech , and V. Guarner‐Lans , “Oxidative Stress, Plant Natural Antioxidants, and Obesity,” International Journal of Molecular Sciences 22 (2021): 1786. 33670130 10.3390/ijms22041786 PMC7916866

[235] P. Zhuang , X. Chen , S. Sun , Y. Li , and H. Mo , “Bioaccessibility and Bioavailability of Pb and Cd in Rice is Affected by Propolis and its Extracts and Fe Intervention,” *The* Science of The Total Environment 951 (2024): 175697. 39182785 10.1016/j.scitotenv.2024.175697

[236] C. Stanton , G. Gardiner , H. Meehan , et al., “Market Potential for Probiotics,” The American Journal of Clinical Nutrition 73 (2001): 476s–483s. 11157361 10.1093/ajcn/73.2.476s

[237] J. A. Ewe , W. N. Wan‐Abdullah , and M. T. Liong , “Viability and Growth Characteristics of Lactobacillus in Soymilk Supplemented With B‐Vitamins,” International Journal of Food Sciences and Nutrition 61 (2010): 87–107. 19961357 10.3109/09637480903334163

[238] T. Halttunen , S. Salminen , and R. Tahvonen , “Rapid Removal of Lead and Cadmium From Water by Specific Lactic Acid Bacteria,” International Journal of Food Microbiology 114 (2007): 30–35. 17184867 10.1016/j.ijfoodmicro.2006.10.040

[239] A. V. Kirillova , A. A. Danilushkina , D. S. Irisov , et al., “Assessment of Resistance and Bioremediation Ability of Lactobacillus Strains to Lead and Cadmium,” International Journal of Microbiology 2017 (2017): 9869145. 28133483 10.1155/2017/9869145 PMC5241453

[240] S. Cheng , “Heavy Metal Pollution in China: Origin, Pattern and Control,” Environmental Science and Pollution Research International 10 (2003): 192–198. 12846382 10.1065/espr2002.11.141.1

[241] V. Singh , G. Ahmed , S. Vedika , et al., “Toxic Heavy Metal Ions Contamination in Water and Their Sustainable Reduction by Eco‐Friendly Methods: Isotherms, Thermodynamics and Kinetics Study,” Scientific Reports 14 (2024): 7595. 38556536 10.1038/s41598-024-58061-3 PMC11365976

[242] A. Karnwal , S. Martolia , A. Dohroo , A. Al‐Tawaha , and T. Malik , “Exploring Bioremediation Strategies for Heavy Metals and POPs Pollution: The Role of Microbes, Plants, and Nanotechnology,” Frontiers in Environmental Science 12 (2024).

[243] H. Zhang , Y. Xu , T. Kanyerere , Y‐s Wang , and M. Sun , “Washing Reagents for Remediating Heavy‐Metal‐Contaminated Soil: A Review,” Frontiers in Earth Science 10 (2022).

[244] A. Ojha , S. Jaiswal , and P. Thakur , S. K. Mishra , “Bioremediation Techniques for Heavy Metal and Metalloid Removal From Polluted Lands: A Review,” International Journal of Environmental Science and Technology 20 (2023): 10591–10612.

[245] A. M. Shackira , J. T. Puthur : Phytostabilization of Heavy Metals: Understanding of Principles and Practices. Plant‐Metal Interactions. In: Edited by Srivastava S , Srivastava AK , Suprasanna P. (Springer International Publishing, 2019): 263–282.

[246] F. S. A. Khan , N. M. Mubarak , M. Khalid , et al., “Magnetic Nanoadsorbents' Potential Route for Heavy Metals Removal‐A Review,” Environmental Science and Pollution Research International 27 (2020): 24342–24356. 32306264 10.1007/s11356-020-08711-6

[247] V. Mohanapriya , R. Sakthivel , N. D. K. Pham , C. K. Cheng , H. S. Le , and T. M. H. Dong , “Nanotechnology‐ A ray of Hope for Heavy Metals Removal,” Chemosphere 311 (2023): 136989. 36309058 10.1016/j.chemosphere.2022.136989

[248] M. Samani , Y. K. Ahlawat , and A. Golchin , “Nano Silica‐Mediated Stabilization of Heavy Metals in Contaminated Soils,” Scientific Reports 14 (2024): 20496. 39227459 10.1038/s41598-024-69182-0 PMC11372104

[249] Y. S. Alia , I. Shaw , C. Chen , Y. Liu : Perspective Chapter: Advanced Nanotechnology Approach for Heavy Metal Toxicity – Analysis, Treatment, and Removal. Heavy Metals in the Environment—Contamination, Risk, and Remediation. In: Edited by Yoshida M (IntechOpen, 2024).

[250] D. B. Olawade , O. Z. Wada , B. I. Egbewole , et al., “Metal and Metal Oxide Nanomaterials for Heavy Metal Remediation: Novel Approaches for Selective, Regenerative, and Scalable Water Treatment,” Frontiers in Nanotechnology 6 (2024).

[251] W. Xia and M. W. King , “Advances in Targeted Delivery of Doxorubicin for Cancer Chemotherapy,” Bioengineering (Basel) 12 (2025): 430. 40281790 10.3390/bioengineering12040430 PMC12025332

[252] Y. Ma , L. Deng , and S. Li , “Application of Nanoparticles in CRISPR/Cas9‐Based Gene Therapy,” Sheng Wu Gong Cheng Xue Bao 38 (2022): 2087–2104. 35786464 10.13345/j.cjb.210739

[253] T. Li , Y. Yang , H. Qi , et al., “CRISPR/Cas9 Therapeutics: Progress and Prospects,” Signal Transduction and Targeted Therapy 8 (2023): 36. 36646687 10.1038/s41392-023-01309-7 PMC9841506

[254] M. Kumar , M. R. Prusty , M. K. Pandey , et al., “Application of CRISPR/Cas9‐Mediated Gene Editing for Abiotic Stress Management in Crop Plants,” Frontiers in Plant Science 14 (2023): 1157678. 37143874 10.3389/fpls.2023.1157678 PMC10153630

[255] F. Liu , R. Li , Z. Zhu , Y. Yang , and F. Lu , “Current Developments of Gene Therapy in Human Diseases,” MedComm 5 (2024): e645. 39156766 10.1002/mco2.645 PMC11329757

[256] H. Mohsin , M. Shafique , M. Zaid , and Y. Rehman , “Microbial Biochemical Pathways of Arsenic Biotransformation and Their Application for Bioremediation,” Folia Microbiologica 68 (2023): 507–535. 37326815 10.1007/s12223-023-01068-6

[257] N. N. Ramli , A. R. Othman , S. B. Kurniawan , S. R. S. Abdullah , and H. A. Hasan , “Metabolic Pathway of Cr(VI) Reduction by Bacteria: A Review,” Microbiological Research 268 (2023): 127288. 36571921 10.1016/j.micres.2022.127288

[258] M. H. Hemmat‐Jou , S. Liu , Y. Liang , G. Chen , L. Fang , and F. Li , “Microbial Arsenic Methylation in Soil‐Water Systems and Its Environmental Significance,”The Science of the Total Environment 944 (2024): 173873. 38879035 10.1016/j.scitotenv.2024.173873

[259] M. M. Naguib , A. O. El‐Gendy , and A. S. Khairalla , “Microbial Diversity of Mer Operon Genes and Their Potential Rules in Mercury Bioremediation and Resistance,” The Open Biotechnology Journal 12 (2018): 56–77.

[260] O. B. Ojuederie and O. O. Babalola , “Microbial and Plant‐Assisted Bioremediation of Heavy Metal Polluted Environments: A Review,” International Journal of Environmental Research and Public Health 14 (2017): 1504. 29207531 10.3390/ijerph14121504 PMC5750922

[261] J. Jaroszuk‐Ściseł , A. Nowak , M. Pac‐Sosińska , D. Kołodyńska , and I. Komaniecka , “Heavy Metal Biosorption Ability of EPS Obtained From Cultures of Fusarium Culmorum Strains With Different Effects on Cereals,” Sustainability 17 (2025): 3744.

